# Phenolate-Pyrazole Ligands in Oxidorhenium(V) Complexes: Catalyst Activation by Chlorido Abstraction

**DOI:** 10.1021/acs.inorgchem.6c01732

**Published:** 2026-05-29

**Authors:** Birgit Ömer, Julia Obermeier, Milan R. Milovanović, Ferdinand Belaj, Nadia C. Mösch-Zanetti, Jörg A. Schachner

**Affiliations:** † Institute of Chemistry, University of Graz, Schubertstraße 1, 8010 Graz, Austria; ‡ Innovation Center of the Faculty of Chemistry, Belgrade, Studentski trg 12-16, 11158 Belgrade, Serbia

## Abstract

The importance of stereoisomers for catalytic perchlorate reduction was first realized with previously published phenol-oxazoline complex [ReOCl­(**L1a**)_2_], where both isomers *N*,*N*-*cis* (**1a**′) and *N*,*N*-*trans* (**1a**) could be separated, proving that the *N*,*N*-*trans* isomer exhibited higher catalytically activity. Similar phenol-pyrazole complexes [ReOCl­(**L2a-e**)_2_] (**2a**′**-e**′) were found to only form the unfavorable *N*,*N*-*cis* isomer, and hence, no catalytic activity in perchlorate reduction was observed. Here, we show that by chlorido abstraction from **2a**′**-e**′ with AgOTf, unexpected isomerization to the *N*,*N*-*trans* cationic complexes [ReO­(**L2a-e**)_2_]­(OTf) (**4a-e**) occurred, resulting in active perchlorate reduction catalysts. Experiments to influence the stereoselectivity by the addition of different pyridine bases were also undertaken. However, no changes in stereoselectivity could be observed. Instead, in one case, a side reaction was observed, giving pyridine adduct [ReOCl_2_(**L2e**)­(py)] (**5e**). The combination of the protic solvent MeOH and electron-withdrawing ligands H**L2d** and H**L2e** also gave side reactions, resulting in the isolation of complexes [ReO­(OMe)­(**L1d**)_2_] (**6d**′) and [ReO­(OMe)­(**L1e**)_2_] (**6e**′). Interestingly, even though these complexes adopt the *N*,*N*-*cis* form in the solid state, they now show some catalytic activity in perchlorate reduction. All novel complexes were fully characterized, for complexes **2b**′, **4b**, **4c**, **5e**, and **6d**′, the solid-state structures were determined by single-crystal X-ray crystallography.

## Graphical abstract

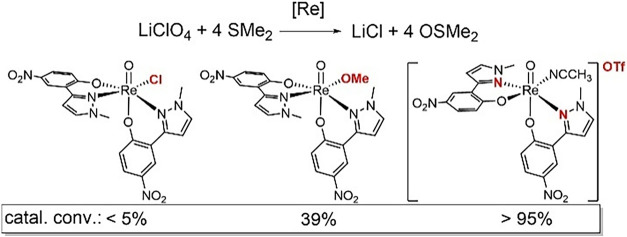

## Introduction

Octahedral complexes of oxidorhodium­(V) belong to a group of coordination complexes that show activity in different, sometimes unusual catalytic transformations. Their unifying structural feature is a single, very short Re=O bond that defines much of their chemistry and catalytic activity. Initially, they had been introduced by the group of Herrmann as a potential alternative, more group-tolerant catalyst for olefin epoxidations compared to methyltrioxorhenium (MTO).^1^ Nowadays, many other catalytic applications have been discovered for oxidorhenium­(V) complexes, including reductions of various functional groups,^2^ organic transformations,^3^ and small molecule activation.^4^ However, in the field of oxygen atom transfer (OAT) catalysis, oxidorhenium­(V) complexes show their most unique activities. There, they belong to a small group of catalysts that show activity in both perchlorate and nitrate reductions under ambient conditions. For a complex of type [ReOX­(ON)_2_], with X being a monodentate and ON being a bidentate ligand, there are in principle six stereoisomers possible (Figure 1).

**Figure 1:**
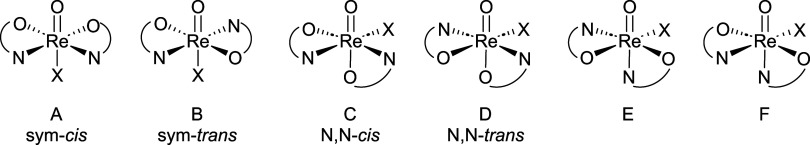
Possible stereoisomers A-F for [ReOX­(ON)_2_] complexes (X = monodentate ligand; ON = bidentate ligand) with the established labeling.

In the year 2000, the group of Abu-Omar disclosed their remarkable findings that oxidorhenium­(V) complex [ReOCl­(**L1a**)_2_] (**1a**) (Scheme 1) is capable to catalytically reduce inert perchlorate to chloride under very mild conditions (25 °C, ambient atmosphere).^5^ In the synthesis of [ReOCl­(**L1a**)_2_], by starting from the well-established precursor [ReOCl_3_(OPPh_3_)­(SMe_2_)] (**P1**), both the *N*,*N*-*cis* (**1a**′) and *N*,*N*-*trans* (**1a**) stereoisomers were formed (Scheme 1).^6^ When using the dimethyloxazoline-phenol ligand H**L1b**, only the *N*,*N*-*trans* complex **1b** is obtained.^6,7^ With both isomers in hand, their catalytic activities were tested, showing that the *N*,*N*-*trans* isomer **1a** is superior in catalytic activity compared to the *N*,*N*-*cis* isomer **1a**′.^6^

**Scheme 1:**
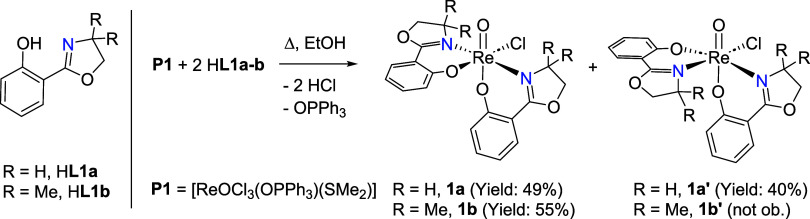
Original Ligand H**L1a** and Synthesis with Yields (In Brackets) of Stereoisomers for Abu-Omar Complex **1a** and Its *N*,*N*-*cis* Isomer **1a**′; Ligand H**L1b** and Stereoselective Synthesis and Yield of Complex **1b** under the Same Abu-Omar Synthesis Conditions (Not Ob. = Not Observed)

Catalytic perchlorate reduction occurs via an oxygen atom transfer (OAT) mechanism using sulfides as a sacrificial oxygen acceptor. Mechanistic studies suggest the initial step to be the loss of the chlorido ligand, generating a vacant coordination site (Scheme 2). The rhenium center cycles between Re­(V) and Re­(VII) during OAT catalysis, and in four consecutive steps, perchlorate is fully reduced to chloride. Oxygen or water (the reaction solvent is a 95/5 vol% mixture of CH_3_CN/H_2_O) was tolerated by the catalyst.^8^ Depending on the isomer that is present, it is the resulting *trans* effect that is responsible for the observed lower catalytic activity of *N*,*N*-*cis* isomers (Scheme 2).^6^

**Scheme 2:**
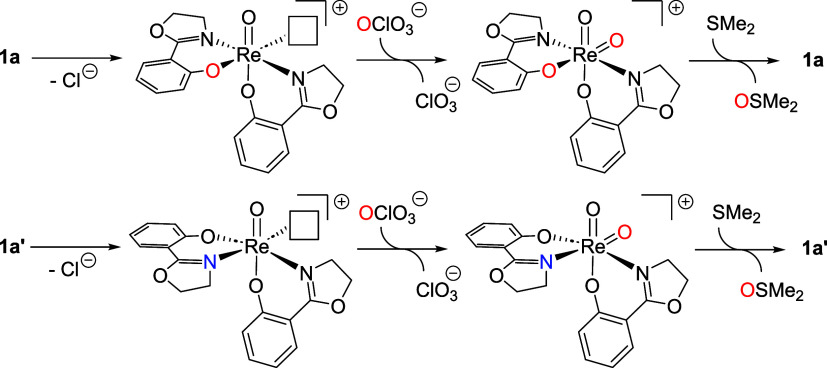
Mechanism of First Step of Catalytic Perchlorate Reduction Exemplified for Both Isomers **1a** and **1a**′^a^

a The stronger *trans* effect of the phenolate oxygen for **1a** compared to the oxazoline nitrogen of **1a**′ is responsible for the observed lower catalytic activity of *N*,*N*-*cis* isomer **1a**′.

This novel understanding triggered the question of how to control the stereoselectivity in the synthesis of these oxidorhenium­(V) complexes. Our solution was a modification of ligand H**L1a**. When two methyl groups were introduced at the oxazoline ring, the resulting ligand H**L1b** (Scheme 1) allowed for the stereoselective synthesis of the *N*,*N*-*trans* isomer of [ReOCl­(**L1b**)_2_] (**1b**).^7^ The catalyst performance of **1b** remained comparable to **1a**. Alternatively, the group of Strathmann found that adding different pyridine bases to the synthesis allowed for isomerization of the less active *N*,*N*-*cis* isomer **1a**′ to the *N*,*N*-*trans* isomer **1a**.^9,10^

We previously reported a set of oxidorhenium­(V) complexes [ReOCl­(**L2a-e**)_2_] (**2a**′**-e**′) containing the phenol-pyrazole ligands H**L2a-e** that were used in olefin epoxidation (Scheme 3).^11,12^ All five complexes adopted the *N*,*N*-*cis* configuration in the solid state (isomer C, Figure 1). Within those studies, complex **2a** was also tested in catalytic perchlorate reduction but was found to be inactive.^11^ In addition, starting from complex **2a**′, the cationic triflate complex [ReO­(**L2a**)_2_]­(OTf) (**4a**, OTf = SO_3_CF_3_^–^) had also been synthesized.^11^ Surprisingly, in the solid state, **4a** had isomerized to the *N*,*N*-*trans* isomer (isomer D, Figure 1). However, at the time of publication, the importance of isomerism for catalytic perchlorate reduction was not yet understood, so cationic *N*,*N*-*trans* complex **4a** was not tested as a catalyst.

**Scheme 3:**
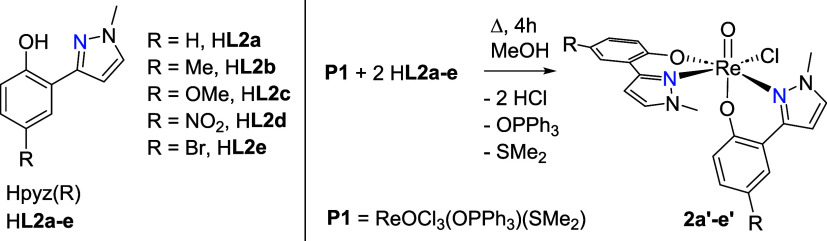
Phenol-Pyrazole Ligands H**L2a-e** (Left); Synthesis of Complexes **2a**′**-e**′ (Right) Resulting in the Exclusive Formation of *N*,*N*-*cis* Isomers

In addition to the isomerization to the *N*,*N*-*trans*-isomer **4a**, in the solid state, the orientation of two **L2a** moieties remained in an asymmetric coordination, with the vacant site at the Re center occupied by a CH_3_CN solvent molecule *cis* to the oxido ligand (Scheme 4, bottom). This overall C_i_-symmetric structure in the solid state differs from the observed symmetry in solution as determined by NMR spectroscopy, which is C_2_-symmetric (Scheme 4, bottom). In contrast, for both phenolate-oxazoline triflate cations [ReO­(**L1a**)]­(OTf) (**3a**) and [ReO­(**L1b**)]­(OTf) (**3b**), the oxazoline ligands remained in the *N*,*N*-*trans* configuration after chlorido abstraction, but an isomerization to a sym-*trans* isomer (Figure 1, isomer B) had occurred (Scheme 4, top). In addition, in the solid state, the sym-*trans* isomer prevailed, and the vacant coordination site was occupied by an adventitious H_2_O molecule *trans* to the oxido ligand.^9,13^ Hence, the solid-state structures for both **3a** and **3b** are in line with their observed NMR spectra in solution. An overview of the isolated and observed stereoisomers depending on the ligand used is shown in Table 1.

**Scheme 4:**
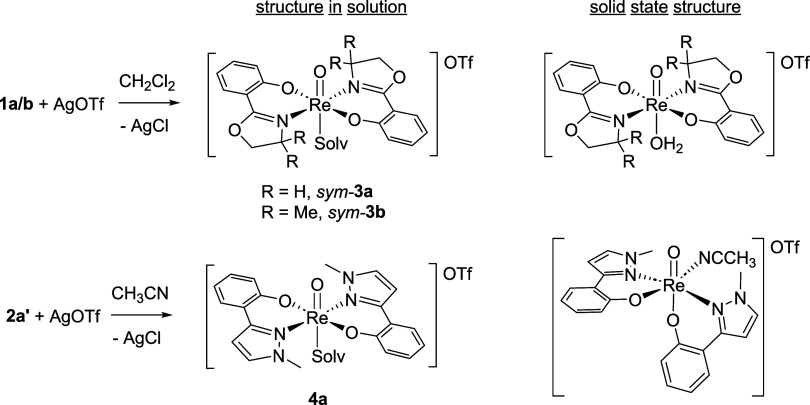
Previously Published Syntheses and Comparisons of Their Solution Structures with Their Obtained Solid-State Structures of Cationic Complexes **3a/b** and **4a**

**Table 1:** Overview of Stereoisomers Observed and Obtained in the Synthesis of Previously Published Complexes **1a/1a**′, **1b**, and **2a**′ and Their Respective Cationic Complexes **3a**, **3b**, and **4a**

ligand	equiv. py(R)	before Cl̅ abstraction	after Cl̅ abstraction	adduct	refs
H**L2a**	0	*N*,*N*-*cis*, **2a**′	*N*,*N*-*trans*, **4a**	CH_3_CN	^11^
H**L1a**	0	*N*,*N*-cis/trans, **1a**′**/1a**			^6^
H**L1a**	5	*N*,*N*-*trans*, **1a**	sym-*trans*, **3a**	H_2_O	^9^
H**L1b**	0	*N*,*N*-*trans*, **1b**	sym-*trans*, **3b**	H_2_O	^13^

Based on the now deeper understanding of the importance of isomerism, we revisited the chemistry of *N*,*N*-*cis* phenol-pyrazole complexes **2a**′**-e**′. Four major questions were addressed: first, are pyridine bases also capable to isomerizes the *N*,*N*-*cis* complexes **2a**′**-e**′ into the desired *N*,*N*-*trans* isomers **2a-e**, as was observed for phenolate-oxazoline complex **1a**′? Second, would those novel complexes **2a-e** now be active catalysts for perchlorate reduction? Third, is the isomerization to the desired *N*,*N*-*trans* isomer after chlorido abstraction also occurring for cationic phenolate-pyrazole complexes **4b-e**, similar to **4a**? Fourth, would the cationic *N*,*N*-*trans* complexes **4a-e** now be catalytically active for perchlorate reduction, in contrast to their chlorido *N*,*N*-*cis* analogues **2a**′**-e**′?

## Results and Discussion

### Stereocontrol by Pyridine Bases on Complexes **2a**′**-e**′

Based on the results of isomerization published by the Strathmann group for *N*,*N*-*cis* complex **1a**′,^9,10^ we also tested three different pyridine bases py­(R) (R = 2,6-H, pyridine; R = 2,6-Me, lutidine; R = 2,6-*t*Bu, BDMEP) in the potential synthesis of the desired *N*,*N*-*trans* complexes **2a-e** (Scheme 5).

**Scheme 5:**
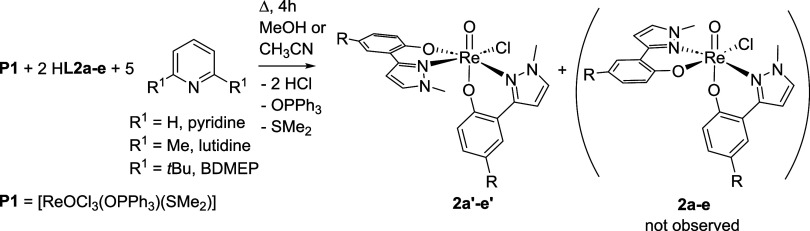
Investigation of Added Pyridine Bases (Pyridine, Lutidine, BDMEP) on the Stereoselectivity of the Synthesis of Complexes **2a**′**-e**′^a^

a No isomerizations to an *N*,*N*-*trans* complex could be observed.

Experiments for the synthesis of complexes **2a**′**-e**′ were performed in either MeOH or CH_3_CN at refluxing temperatures for four hours. Five molar equivalents of either pyridine, lutidine, or BDMEP were added. After cool down, an aliquot of the crude reaction mixture was analyzed by ^1^H NMR spectroscopy. In none of the spectra, *N*,*N*-*trans* complexes could be detected (SI, Tables S1–S5). However, from the reaction with Hpyz­(NO_2_) (H**L2e**), a few green crystals suitable for X-ray diffraction analysis could be isolated. The structural analysis (SI, Figure S25, and Tables S6–S7) revealed the formation of [ReOCl_2_(**L2e**)­(py)] (**5e**), with a coordinated pyridine (Scheme 6). Presumably, after initial coordination of one equivalent of ligand **L2e**, the weaker Lewis base OPPh_3_ in the nonobserved complex {ReOCl_2_(**L2e**)­(OPPh_3_)} was substituted by the stronger Lewis base pyridine to give **5e**.

**Scheme 6:**
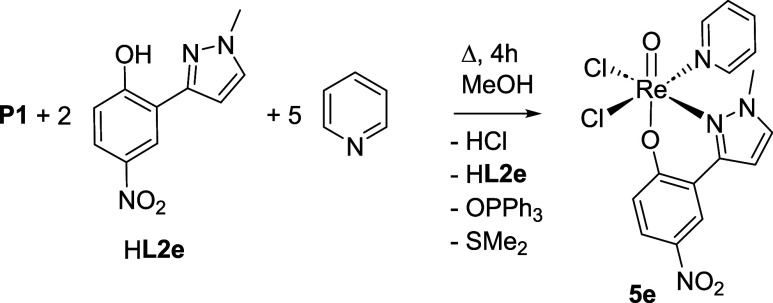
Unexpected Formation of Mono-Ligated Pyridine Complex **5e**

In the reaction with the electron-withdrawing ligands HpyzBr (H**L2d**) and Hpyz­(NO_2_) (H**L2e**), respectively, in the presence of lutidine in MeOH, next to the respective N,N-*cis* complex **2d**′ or **2e**′, side products could be identified by NMR spectroscopy in the form of methoxide complexes [ReO­(OMe)­(**L2d**)_2_] (**6d**′) and [ReO­(OMe)­(**L2e**)_2_] (**6e**′) (Scheme 7), respectively. For complex **6d**′, a few single crystals suitable for X-ray diffraction analysis (Figure 4, right) could be separated from the reaction mixture, while all attempts to synthesize **6d**′ in significant amounts failed. In the solid state, **6d**′ also adopts the *N*,*N*-*cis* conformation (right, Figure 3). It seems that after formation of **2d**′, a follow-up reaction with the solvent MeOH under elimination of HCl occurred, supported by the base lutidine. In contrast to **6d**′, complex **6e**′ had formed in larger amounts as a side product. Pure material of **6e**′ could be obtained by washing the reaction mixture repeatedly with small amounts of MeOH, which left behind the less soluble complex **2e**′. However, single crystals of **6e**′ of high enough quality for X-ray diffraction analysis could not be obtained.

**Scheme 7:**
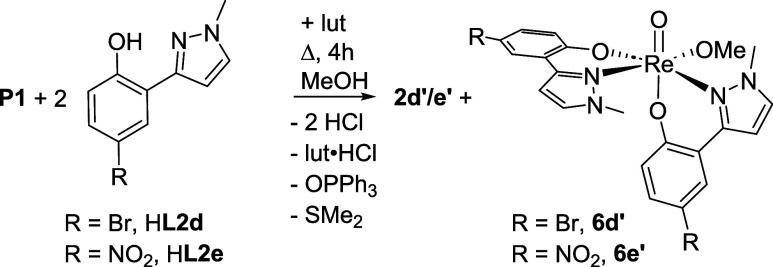
Reaction with Electron-Withdrawing Ligands H**L2d/e** in MeOH with Lutidine (= 2,6-Dimethylpyridine, Lut)

Lastly, the sterically most hindered pyridine base BDMEP seemed to have no influence at all, either on stereocontrol or in causing side reactions. Similar observations for BDMEP were also made by the group of Strathmann and co-workers.^9,10^

### Synthesis of Cationic Complexes **4b-e**

The syntheses of cationic triflate complexes **4b-e** were performed under similar conditions to complex **4a**, which was previously reported.^5^ Thus, in an acetonitrile solution, one equivalent of AgOTf was added to the respective starting complex **2b**′**-e**′. Heating to refluxing conditions for one hour led to high conversions (>85% yield). A color change from green to yellow-greenish was observed upon abstraction of the chlorido ligand. All four novel complexes **4b-e** only show one set of ligand signals in NMR spectroscopy (see SI), perceptively with a higher symmetry than expected from the solid-state structures (Figure 4). The same behavior was observed for complex **4a**, where at 25 °C, a time-averaged C_2_-symmetric species was observed due to fast molecular dynamics.^11^ It is feasible that the same isomerism is also occurring for complexes **4b-e**, respectively. In order to elucidate the isomeric conformation in solution, a 2D-NOESY experiment on complex **4d** was performed (Figure 2). Indeed, a cross-peak from the *N*-methyl group (at 4.24 ppm) to an *ortho* proton (at 8.26 ppm) of a phenolate ring could be observed (Figure S24), supporting that the two ligand moieties remain in the *N*,*N*-*trans* form in complex **4d** in solution.

**Figure 2:**
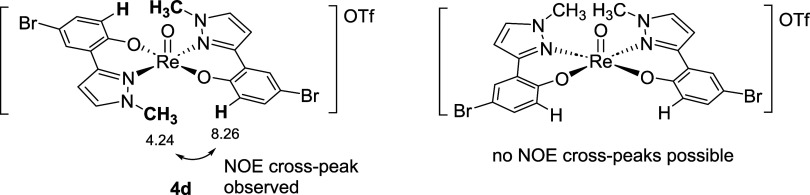
Two potential solution structures for symmetric complex **4d** as a sym-*trans* (C_2_, left) or sym-*cis* (C_s_, right). The observed NOE between the methyl group and the *ortho* proton confirmed the sym-*trans* isomer of **4d** in solution.

By abstraction of the chlorido ligand, a five-coordinate intermediate species like **Int1** may be formed, which rearranges to a trigonal-bipyramidal coordination polyhedron (Scheme 8). Because of the strong *trans*-directing influence of the oxido ligand, a second rearrangement to square-pyramidal intermediate **Int2** occurs, either leading to C_S_-symmetric isomer *cis*-**Int2 or** C_2_-symmetric isomer *trans-* **Int2**. Indeed, in the NMR spectra of cationic complexes **4a-e**, only one set of ligand signals are observed, showing a higher symmetry than C_1_-symmetric complexes **2a**′**-e**′. Again, based on the *trans*-directing influence of oxygen-containing ligands, isomer *trans*-**Int2**, with the phenolate oxygen atoms *trans* to each other, should be the thermodynamically favored isomer over *cis*-**Int2**, potentially explaining why only one symmetric species is observed by ^1^H NMR spectroscopy after chlorido abstraction. In two cases, for complexes of type [ReOCl­(ON)_2_], the potential energies of the six possible isomers A-F (Figure 1) were calculated, showing both times that the *N*,*N*-*trans* isomer is the most stable one.^14^ However, with the analytical data at hand, the true mechanism of this isomerization cannot be finally answered with certainty.

**Scheme 8:**
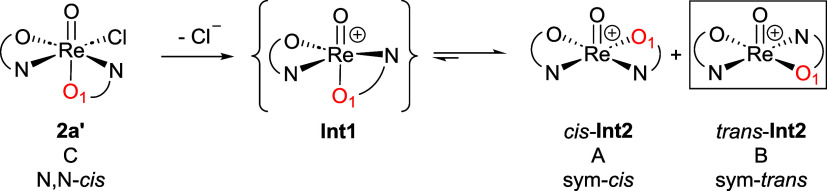
Suggested Mechanism for the Stereoselective Isomerization to Yield C_2_-Symmetric *trans-* **Int2** (Boxed) over C_s_-Symmetric Species *cis-* **Int2** via Trigonal-Bipyramidal Intermediate **Int1** after Chlorido Abstraction from **2a**′

### Molecular Structure

Single crystals of [ReOCl­(**L2b**)_2_] (**2b**′) were obtained from a concentrated solution in MeOH at 8 °C. The solid-state structure confirms the *N*,*N*-*cis* configuration of **2b**′ (Figure 3, left). The rhenium center is coordinated in a distorted octahedral fashion. For example, the O1-Re1-O2 angle is only at 163.32(6)°. All bond lengths and angles are within the expected range, and selected ones are given in Table 2. Single crystals of [ReO­(OMe)­(**L2d**)_2_] (**6d**′) were obtained from a concentrated solution in CH_2_Cl_2_ layered with heptane at 8 °C. The complex also adopts an *N*,*N*-*cis* configuration, with the methoxide ligand completing the distorted octahedral coordination sphere. For example, the O1-Re1-O2 angle is only at 164.54(15)°. Selected bond distances are shown in Table 2.

**Figure 3:**
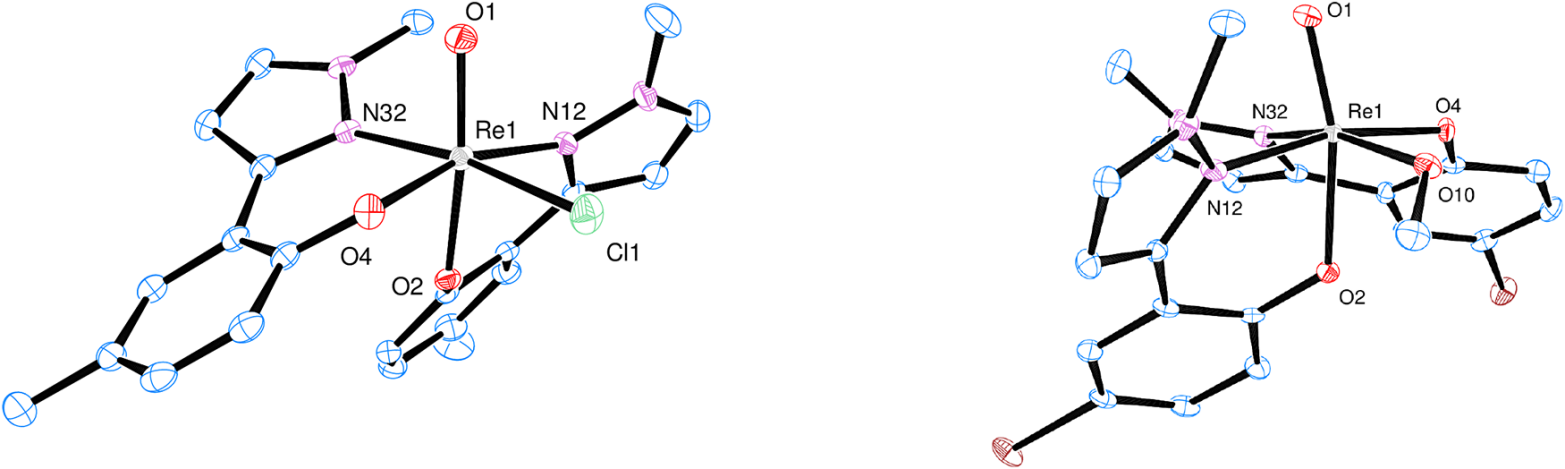
Molecular views (50% level) of complexes [ReOCl­(**L2b**)_2_] (**2b**′, left) and [ReO­(OMe)­(**L2d**)_2_] (**6d**′, right) (H atoms omitted for clarity).

**Table 2:** Selected Bond Lengths [Å] of **2b**′, **4b**, **4c**, and **6d**′

	Re=O1	Re–O2	Re–O4	Re–N12	Re–N32	Re–X
**2b**′	1.6943(15)	1.9836(14)	1.9725(14)	2.1221(16)	2.1340(17)	2.3617(5), X = Cl1
**4b**	1.680(3)	1.959(3)	1.963(3)	2.113(4)	2.080(4)	2.144(4), X = N1
**4c**	1.695(2)	1.950(2)	1.948(2)	2.076(3)	2.130(3)	2.161(3), X = N1
**6d**′	1.695(3)	2.026(3)	1.988(3)	2.124(4)	2.127(4)	1.954(3), X = O10

Single crystals of cationic complexes [ReO­(CH_3_CN)­(**L2b**)_2_]­OTf (**4b**) and [ReO­(CH_3_CN)­(**L2c**)_2_]­OTf (**4c**) were obtained from a concentrated solution in CH_3_CN at 8 °C, respectively. The solid-state structures each confirm their *N*,*N*-*trans* configuration. For both complexes, in the solid state, a CH_3_CN molecule is coordinated as the sixth ligand to the rhenium center *cis* to the oxido ligand, completing a distorted octahedral coordination sphere. For example, the O1-Re1-O2 angles for **4b** and **4c** are 164.22(13)° and 163.26(10)°, respectively. Selected bond distances are shown in Table 2.

Interestingly, by the data shown in Table 2, the solid-state structural parameters of complexes **2b**′, **4b**, **4c**, and **6d**′ do not vary significantly from each other. Seemingly, there is no influence of overall charge, isomeric conformation or nature of ligand X on the respective bond lengths around the Re center (Figure 4).

**Figure 4:**
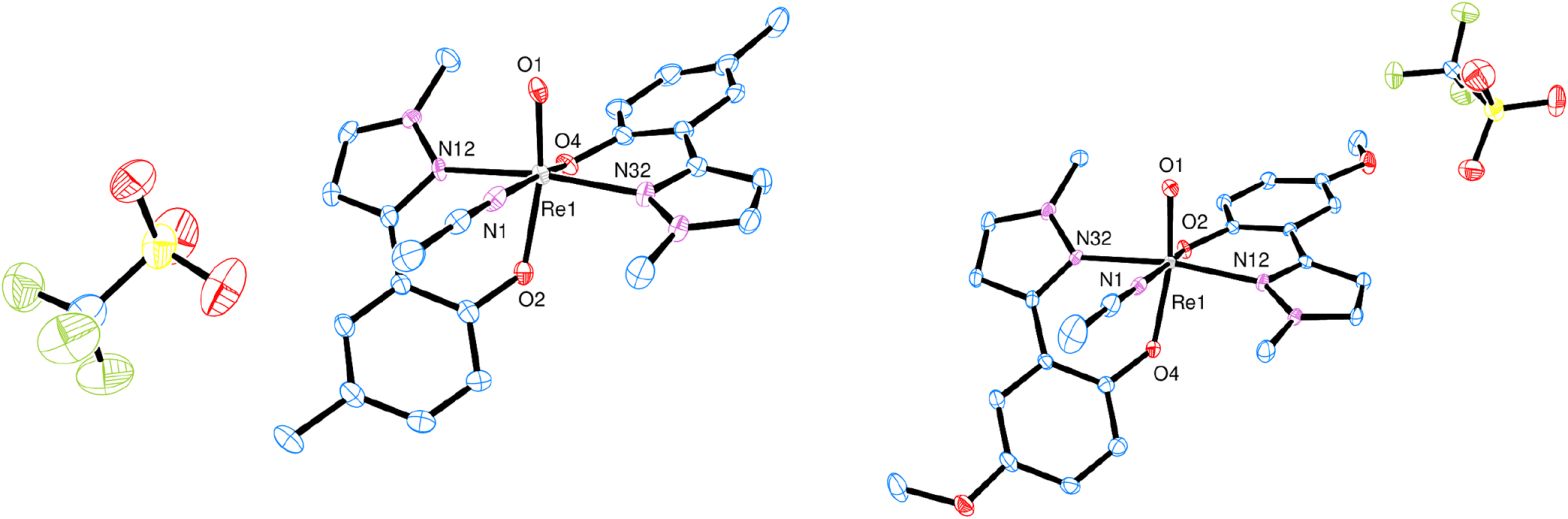
Molecular views (50% level) of complexes [ReO­(CH_3_CN)­(**L2b**)_2_]­OTf (**4b**, left) and [ReO­(CH_3_CN)­(**L2c**)_2_]­OTf (**4c**, right) (H atoms omitted for clarity).

### Investigation of Catalytic Properties of Novel Complexes

First, we repeated the perchlorate reduction experiments with all *N*,*N*-*cis* chlorido complexes **2a**′**-e**′ to also confirm the inactivities of previously untested complexes **2b**′**-e**′. All catalytic experiments were performed with a 10 mol% catalyst loading, four equivalents of SMe_2_ (DMS) as sacrificial oxygen acceptor in a 95/5 vol% mixture of CD_3_CN/D_2_O at room temperature. The progress of the catalytic reaction was followed by ^1^H NMR spectroscopy via monitoring the conversion of DMS to DMSO over 24 h. As expected, no catalytic activity for **2a**′**-e**′ could be observed (Table 3). Next, the cationic *N*,*N*-*trans* complexes **4a-e** were tested under the same conditions, and we were delighted to observe high conversions between 89% and >95% of DMS to DMSO, proving their catalytic activity (Table 3). Out of curiosity, we also tested side product [ReO­(OMe)­(**L2e**)_2_] (**6e**′) and were surprised to observe a 39% conversion to DMSO. Even though we could not obtain the solid-state structure of **6e**′ based on the *N*,*N*-*cis* isomer of related complex **6d**′, it is safe to assume that **6e**′ also adopts the *N*,*N*-*cis* configuration. It seems that the methoxido ligand in **6e**′ is less tightly bound than the chlorido ligand in **2e**′, still allowing to initiate the catalytic cycle by dissociation from the rhenium center. The overall lower activity of **6e**′ compared to **4e** is then still due to being the “wrong” isomer of *N*,*N*-*cis*.

**Table 3:** Overview of Perchlorate Reduction Catalysis Results^a^

		Conv. to DMSO [%]^b^		Conv. to DMSO [%]^b^
1	**2a**′	<5	**4a**	92
2	**2b**′	<5	**4b**	>95
3	**2c**′	<5	**4c**	>95
4	**2d**′	<5	**4d**	89
5	**2e**′	<5	**4e**	>95
6	**6e**′	39		

a General conditions: 10 mol % catalyst, 4 equiv of SMe_2_ (DMS), 95/5 vol% CD_3_CN/D_2_O, rt.

b Conversion of DMS to DMSO after 24 h.

Based on the results of catalytic activities presented in Table 3, a dramatic increase is apparent. Based on this data, two different positive effects could be responsible: the isomerization to *N*,*N-trans* and/or the generation of a vacant coordination site by the triflate anion. In 2014, we could isolate both isomers of Abu-Omar’s complex [ReOCl­(oz)_2_], the *N*,*N*-*trans* isomer **1a** and the *N*,*N*-*cis* isomer **1a**′, and test them individually in perchlorate reduction under identical conditions.^6^ We found that the *N*,*N*-*trans* isomer **1a** was superior in catalytic activity compared to *N*,*N*-*cis* **1a**′. DFT calculations showed that the redox catalysis, where the rhenium cycles between Re­(V) and Re­(VII) back to Re­(V), is thermodynamically less demanding compared to the *N*,*N*-*cis* isomer **1a**′. We could also show that even the triflate cation of *N*,*N*-*cis* complex **1a**′ is still less catalytically active compared to the *N*,*N*-*trans* chlorido complex **1a**. While there are numerous examples of enhanced catalytic activity by switching to a weakly coordinating anion, we believe that in this case, the adoption of the *N*,*N*-*trans* isomeric form is the main contributor to the enhanced catalytic activity of complexes **4a-e**. But of course, also the triflate cation will contribute to a certain extent. To what amount is currently unknown, though.

In summary, it can be assumed that all five cationic triflate complexes **4a-e** do isomerize to the *N*,*N*-*trans* isomer. Together with the now vacant coordination site, enabled by the weakly coordinating anion OTf, complexes **4a-e** show greatly increased catalytic activities. Considering that a majority of the published [ReOCl­(ON)_2_] complexes adopt the *N*,*N*-*cis* isomeric form,^15^ it would be interesting to test if other published *N*,*N*-*cis* isomers would undergo isomerization upon chlorido abstraction.

## Conclusions

Within this manuscript, we describe a novel way to activate the otherwise inactive, neutral N,N-*cis* complexes **2a**′**-e**′ for catalytic perchlorate reduction by chlorido abstraction with AgOTf. The obtained cationic complexes **4a-e** isomerize to the *N*,*N*-*trans* isomers, thereby becoming active for catalytic perchlorate reduction. The reason for this isomerization is most likely due to the *trans*-directing properties of oxygen ligands (oxido and phenolate oxygen). This observation leads to a new set of catalysts for the challenging reduction of perchlorate inside the family of oxidorhenium­(V) [ReOX­(ON)_2_] catalysts. Furthermore, the unexpected catalytic activity of methoxido *N*,*N*-*cis* complex **6e**′ leads to another interesting entry point of potential novel catalysts, by changing the anionic monodentate ligand X in catalysts of the general structure [ReOX­(ON)_2_]. We are currently exploring the full potential of novel catalyst **4a-e** in perchlorate reduction and will also start investigations for the related nitrate reduction catalysis.

## Experimental Section

### General

The rhenium precursor [ReOCl_3_(OPPh_3_)­(SMe_2_)] (**P1**) was prepared according to previously published methods.^16^ Full synthesis details via the novel enamine route of H**L2a-e** can be found in the SI. Chemicals were purchased from commercial sources and used without further purification. NMR spectra were recorded with a Bruker Avance (300 MHz) instrument. Chemical shifts are given in ppm and are referenced to residual protons in the solvent. Coupling constants (J) are given in Hertz (Hz). HR-MS (ESI) measurements were recorded with an Agilent Technologies 6230 TOF LC/MS with ESI mass spectrometer in positive ion mode, and the used solvent was acetonitrile. Peaks are denoted as cationic mass peaks, and the unit is according to the ion mass/charge ratio. Samples for infrared spectroscopy were measured on a Bruker Optics α FT-IR spectrometer equipped with an ATR diamond probe head. GC-MS measurements were performed on an Agilent 7890 A with an Agilent 19091J–433 column coupled to a mass spectrometer-type Agilent 5975 C.

Details regarding the modified synthesis of ligands H**L2a-e**, isomerization experiments with pyridine bases of complexes **2a**′**-e**′, NMR data of novel compounds, as well as on X-ray data collection are available within the article and its Supporting Information (SI). Metadata is deposited at Zenodo under the DOI 10.5281/zenodo.17954864. CCDC numbers 2468748–2468752 contain the supplementary crystallographic data for this paper. This data can be obtained free of charge via http://www.ccdc.cam.ac.uk/ or from Cambridge Crystallographic Data Centre, 12 Union Road, Cambridge, CB2 1EZ, UK.

#### Synthesis of [ReO­(**L2b**)_2_]­OTf (**4b**)

0.35 mmol (1 equiv.; 220 mg) of **2b**′ were mixed with 0.39 mmol (1.1 equiv.; 100 mg) of AgOTf in 20 mL of dry CH_3_CN. The greenish suspension was heated to refluxing conditions for 1 h, under which a white precipitate of AgCl and a reddish reaction solution formed. The solution was filtered hot, and upon slow cooling, crystals of **4b** were formed in quantitative yield (96% yield; 247 mg, 0.34 mmol). ^1^H NMR (300 MHz, Acetonitrile-*d*_3_) δ 8.20 (d, *J* = 2.8 Hz, 2H, ar), 7.79 (d, *J* = 2.2 Hz, 2H, ar), 7.29–7.18 (m, 4H, ar), 6.92 (d, *J* = 8.3 Hz, 2H, ar), 4.22 (s, 6H, N-C*H*_*3*_), 2.41 (s, 6H, C_ar_-CH_3_). ^13^C NMR (HSQC, 75 MHz, Acetonitrile-*d*_3_) δ 140.93, 133.00, 129.18, 118.71, 104.76 (all ar), 41.38 (N-*C*H_3_), 20.59 (C_ar_-CH_3_) (some C-atoms are obscured due to low solubility of the complex). ATR-IR (cm^–1^): 3122 (w), 2926 (w), 2829 (w), 1515 (C=N, m), 1489 (m), 1465 (m), 1417 (m), 1280 (m), 1207 (s), 1157 (s), 1021(s), 956 (m, Re=O), 872 (m), 802 (m), 633 (s), 531 (m), 513 (m); UV–vis (CH_3_CN) λ_max_, nm (ε): 655 (94); HR-ESI-MS (pos, *m*/*z*): calc. for {**3b** ^+^} = 577.12494, obs. = 577.1292.

#### Synthesis of [ReO­(**L2c**)_2_]­OTf (**4c**)

0.40 mmol (1 equiv.; 258 mg) of **2c**′ were mixed with 0.44 mmol (1.1 equiv.; 110 mg) of AgOTf in 20 mL of dry CH_3_CN. The greenish suspension was heated to refluxing conditions for 1 h, under which a white precipitate of AgCl and a dark red-brownish reaction solution formed. The solution was filtered hot, and upon slow cooling to 8 °C, crystals of **4c** were formed in almost quantitative yield (95% yield; 288 mg, 0.38 mmol). ^1^H NMR (300 MHz, Acetonitrile-*d*_3_) δ 8.21 (d, *J* = 2.9 Hz, 2H, ar), 7.44 (d, *J* = 2.7 Hz, 2H, ar), 7.26 (d, *J* = 2.9 Hz, 2H, ar), 7.06–6.92 (m, 4H, ar), 4.23 (s, 6H, N-C*H*_*3*_), 3.86 (s, 6H, C_ar_-OC*H*_*3*_). ^13^C NMR (HSQC, 75 MHz, Acetonitrile-*d*_3_) δ 141.22, 119.64, 112.63, 105.56 (all ar), 56.20 (C_ar_-O*C*H_3_), 41.10 (N-*C*H_3_) (some C-atoms are obscured due to low solubility of the complex). ATR-IR (cm^–1^): 3145 (w), 3113 (w), 2920 (w), 2853 (w), 1516 (C=N, m), 1488 (m), 1467 (m), 1406 (m), 1258 (s), 1234 (s), 1221 (s), 1147 (s), 1097 (m), 1026 (s), 954 (m, Re=O), 870 (m), 808 (m), 783 (m), 631 (s), 602 (m), 554 (m), 514 (m); UV–vis (CH_3_CN) λ_max_, nm (ε): 389 (89). HR-ESI-MS (pos, *m*/*z*): calc. for {**4c** ^+^} = 609.11477, obs. = 609.1164.

#### Synthesis of [ReO­(**L2d**)_2_]­OTf (**4d**)

0.42 mmol (1 equiv.; 312 mg) of **2d**′ were mixed with 0.46 mmol (1.1 equiv.; 120 mg) of AgOTf in 20 mL of dry CH_3_CN. The greenish suspension was heated to refluxing conditions for 4 h, under which a white precipitate of AgCl and a dark brownish reaction solution formed. The solution was filtered hot, and upon slow cooling to 8 °C, crystals of **4d** were formed in almost quantitative yield (91% yield; 342 mg, 0.38 mmol). ^1^H NMR (300 MHz, Acetonitrile-*d*_3_) δ 8.24 (d, *J* = 2.9 Hz, 2H, ar), 8.13 (d, *J* = 2.5 Hz, 2H, ar), 7.51 (dd, *J* = 8.8, 2.5 Hz, 2H, ar), 7.26 (d, *J* = 2.9 Hz, 2H, ar), 6.95 (d, *J* = 8.8 Hz, 2H, ar), 4.21 (s, 6H, N-C*H*_*3*_); ^13^C NMR (75 MHz, Acetonitrile-*d*_3_) δ 149.35, 141.98, 135.42, 131.76, 121.88, 114.67, 105.96 (all ar), 41.60 (N-*C*H_3_) (some C-atoms are obscured due to low solubility of the complex); ATR-IR (cm^–1^): 3146 (w), 2994 (w), 2930 (w), 2320 (w), 2287 (w), 1512 (w), 1251 (s, C=N), 1220 (s, C=N), 1158 (s), 1022 (s), 953 (Re=O, m), 732 (m), 624 (s), 513 (m); UV–vis (CH_3_CN) λ_max_, nm (ε): 555 (110); HR-ESI-MS (pos, *m*/*z*): calc. for {**4e** ^+^} = 704.91467, obs. = 704.9146.

#### Synthesis of [ReO­(**L2e**)_2_]­OTf (**4e**)

0.43 mmol (1 equiv.; 290 mg) of **2d’** were mixed with 0.47 mmol (1.1 equiv.; 121 mg) of AgOTf in 20 mL of CH_3_CN. The greenish suspension was heated to refluxing conditions for 4 h, under which a white precipitate of AgCl and a dark brownish reaction solution formed. The solution was filtered hot, and upon slow cooling to 8 °C, crystals of **4d** were formed in almost quantitative yield (85% yield; 303 mg, 0.37 mmol). ^1^H NMR (300 MHz, Acetonitrile-*d*_3_) δ 8.87 (d, *J* = 2.8 Hz, 2H, ar), 8.32 (d, *J* = 2.8 Hz, 2H, ar), 8.26 (dd, *J* = 9.1, 2.9 Hz, 2H, ar), 7.46 (d, *J* = 2.9 Hz, 2H, ar), 7.17 (d, *J* = 9.1 Hz, 2H, ar), 4.24 (s, 6H, N-C*H*_*3*_). ^13^C NMR (HSQC, 75 MHz, Acetonitrile-*d*_3_) δ 125.99 (ar), 41.73 (N-*C*H_3_) (all other C-atoms are obscured due to low solubility of the complex); ATR-IR (cm^–1^): 3133 (w), 2923 (w), 2851 (w), 1604 (w), 1571 (w), 1500 (s), 1337 (s, C = N), 1315 (s, C = N), 1282 (s, C = N), 1256 (s, C=N), 1128 (m), 963 (s, Re=O), 902 (m), 879 (s), 783 (s), 735 (s), 673 (m), 653 (m), 424 (m); UV–vis (CH_3_CN) λ_max_, nm (ε): 540 (108); HR-ESI-MS (pos, *m*/*z*): calc. for {**4d** ^+^} = 639.06380, obs. = 639.0681.

## Supplementary Material



## Data Availability

Metadata is deposited at Zenodo under the DOI 10.5281/zenodo.17954864.

## Notes

Details of synthesis of ligands H**L2a-d** via the enamine route (**e1a-d**) and for ligand H**L2e**; depiction of ^1^H NMR spectra of H**L2a-e** and side products H**L2a**′**-e**′, ^1^H and ^13^C NMR (HSQC) spectra of novel triflate complexes **4b-e**; ^1^H NMR spectrum of **6e**′; 2D-NOESY spectrum of **4d**; overview of results of isomerization experiments with three different pyridine bases (Tables S1–S5) (PDF)
