# Supplementary material for: Phenolate-Pyrazole Ligands in Oxidorhenium(V) Complexes: Catalyst Activation by Chlorido Abstraction
Source: Inorg Chem. 2026 May 29;65(23):13198–205. doi: 10.1021/acs.inorgchem.6c01732 (PMC13273805; doi:10.1021/acs.inorgchem.6c01732)
Supplement: Supplementary file 1 [file ic6c01732_si_001.pdf]

Supporting Information for

Phenolate-pyrazole ligands in oxidorhenium(V) complexes: catalyst activation by chlorido abstraction

Birgit Ömer,† Julia Obermeier,† Milan R. Milovanović,†,‡ Ferdinand Belaj,† Nadia C. Mösch-Zanetti† and Jörg A. Schachner†,*

†Institute of Chemistry, University of Graz, Schubertstraße 1, 8010 Graz, Austria.

‡Innovation Center of the Faculty of Chemistry, Belgrade, Studentski trg 12-16, 11158 Belgrade, Serbia.

corresponding author: joerg.schachner@uni-graz.at

Improved synthesis of ligands HL2a-e. Previously, we had synthesized the phenol-pyrazole ligand **HL2** from the corresponding 2-hydroxy-acetophenons by deprotonation with NaH (60% in mineral oil) and reacting with an excess of ethylformate to obtain the respective β-ketoaldehyde. Ring-closure to give the pyrazole was then achieved by reaction with methylhydrazine under heating.[1,2] Yields varied between 42 and 75% and were sometimes difficult to achieve consistently. A higher yielding, more atom-efficient synthesis is described here now, using N,N-dimethylformamide dimethylacetal (DMF·DMA) to obtain the corresponding enamins **e2a-d**.[3] DMF·DMA is condensed in equimolar amounts with the respective 2-hydroxy-acetophenon under neat conditions, giving > 95% yields of the enamine (Scheme S1). Work-up therefore is not needed, and the crude enamines can be used directly for the next step of ring-closure with methyl-hydrazine. Nitro-substituted ligand **HL2e** is more conveniently prepared from commercially available 6-nitrochromone in a single step, so the two-step synthesis via an enamine was not investigated.

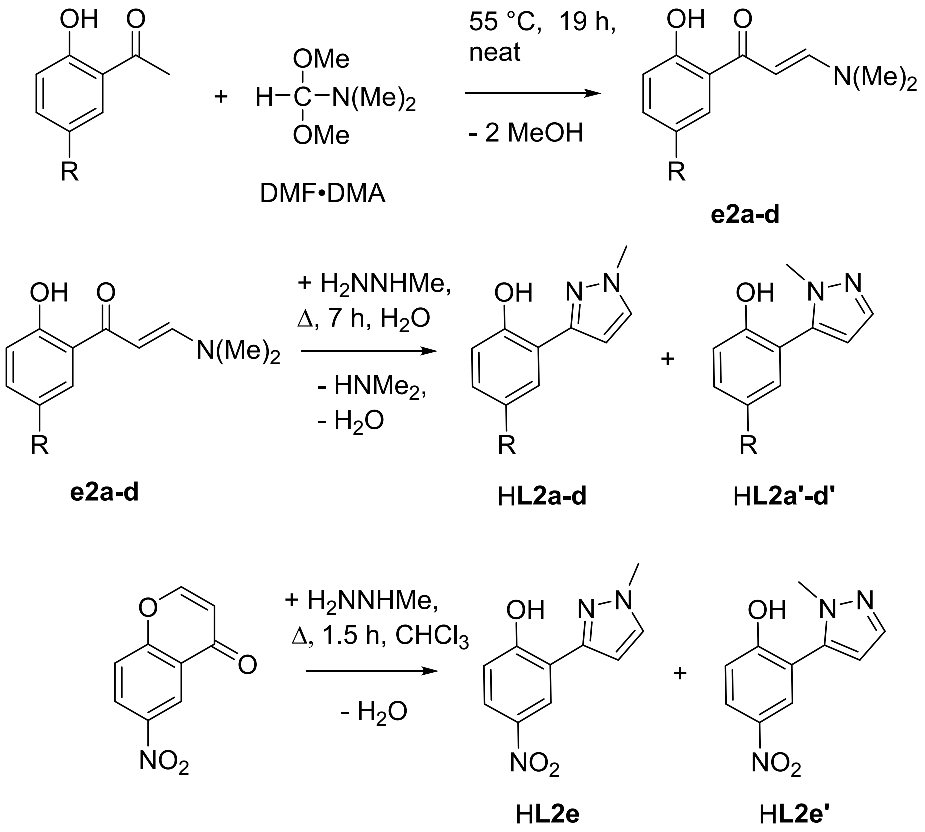

Scheme S1. Improved synthesis of ligands HL2a-d via enamines e2a-d (R = H, e2a; R = Me, e2b; R = OMe, e2c; R = Br, e2d); One-step synthesis of HL2e from commercially available 6-nitrochromone.

In the ring-closing reaction with methylhydrazine, besides the targeted ligands HL2a-e, also the side products HL2a'-e' are obtained, with the methyl group on N1 in the pyrazole ring (**Error! Reference source not found.**). Although several reaction conditions were tested and some strategies are described in literature[4] to minimize formation of HL2a'-e', no satisfying procedure was found, ultimately requiring column chromatography to separate HL2a-e from HL2a'-e'. Usually, a 75-85% ratio of the desired ligands HL2a-e was achieved. The unwanted isomers HL2a'-e' can be easily identified in ^{1}H NMR spectroscopy by their unusually high up-field shifted phenolic -OH proton around 5 ppm (see SI).

Detailed synthesis of ligands

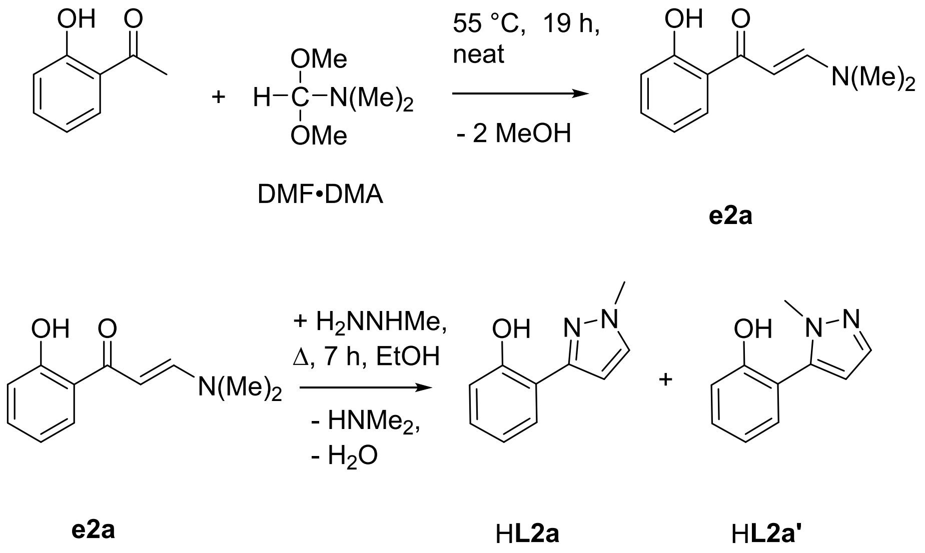

Scheme S2. Synthesis of H**L2a** via enamine **e2a**.

Synthesis of **e2a**. 5.000 g (1 equiv., 36.72 mmol) 2′-Hydroxyacetophenon and 4.889 ml (1 equiv.; 36.72 mmol) N,N-dimethylformamide-dimethylacetal (DMF·DMA) were heated under neat conditions to 55 °C for 19 h. The resulting deep red solution was evacuated to dryness to give 6.88 g of **e2a** (98%, 36 mmol). TLC (EtOAc/cyHex 1/1): 0.31; ^{1}H NMR (300 MHz, Chloroform-d) δ 13.91 (s, 1H), 7.89 (d, J = 12.2 Hz, 1H), 7.70 (dd, J = 8.0, 1.7 Hz, 1H), 7.35 (ddd, J = 8.6, 7.2, 1.6 Hz, 1H), 6.93 (dd, J = 8.3, 1.2 Hz, 1H), 6.82 (ddd, J = 8.2, 7.1, 1.2 Hz, 1H), 5.79 (d, J = 12.2 Hz, 1H), 3.20 (s, 3H), 2.98 (s, 3H). The analytical data matches published literature.[3,5]

Synthesis of H**L2a**. 0.50 g (1 equiv., 2.63 mmol) of **e2a** were dissolved in 10 mL EtOH and 0.152 mL (1.1 equiv., 2.89 mmol) methylhydrazine were added. The red reaction solution was heated to refluxing temperatures for 4 h. After cooling down to rt, the reaction was evacuated to dryness, and aqueous work-up with CH_2Cl_2 (3 x 20 mL) yielded a bright-orange mixture of H**L2a** and H**L2a'**. After column chromatography (EtOAc/cyHex 1/1), 245.6 mg of H**L2a** were obtained as a bright yellow solid (53%, 1.41 mmol). TLC (EtOAc/cyHex 1/1): H**L2a** = 0.40. Other analytical data matches published literature.[1]

Analytical data for H**L2a'**. TLC (EtOAc/cyHex 1/1): 0.18; ^{1}H NMR (300 MHz, Chloroform-d) δ 10.80 (s, 1H), 7.61 (d, *J* = 1.8 Hz, 1H), 7.35 (td, *J* = 7.7, 1.7 Hz, 1H), 7.20 (dd, *J* = 7.8, 1.7 Hz, 1H), 7.05 – 6.97 (m, 2H), 6.36 (d, *J* = 1.9 Hz, 1H), 5.36 (s, 1H), 3.79 (s, 3H). ^{1}H NMR (300 MHz, Acetonitrile-d3) δ 14.29 (s, 1H), 7.91 (d, *J* = 12.0 Hz, 1H), 7.83 (dd, *J* =

8.2, 1.7 Hz, 1H), 7.35 (m, 1H), 6.87 – 6.79 (m, 2H), 5.87 (d, *J* = 12.0 Hz, 1H), 3.18 (s, 3H), 2.97 (s, 3H).

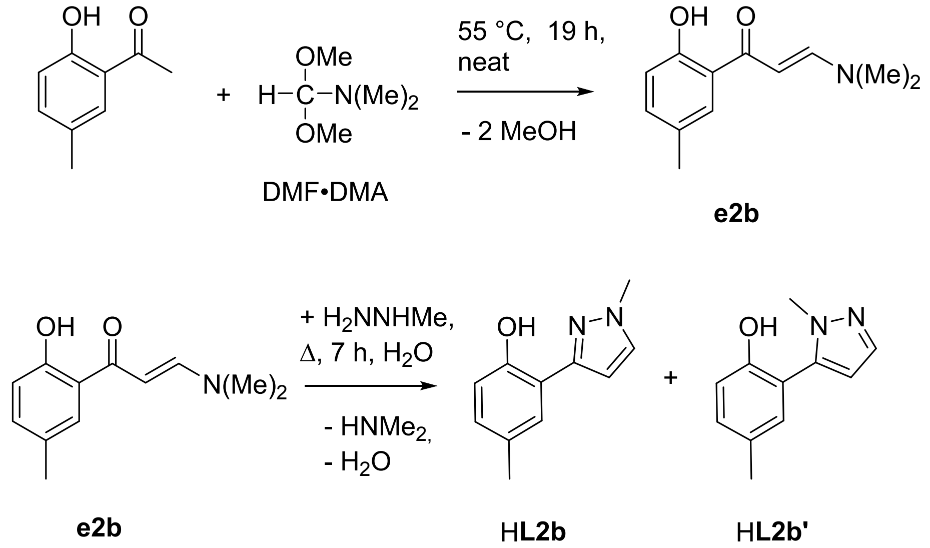

Scheme S3. Synthesis of H**L2b** via enamine **e2b**.

Synthesis of **e2b**. 5.03 g (1 equiv., 33.4 mmol) 2′-Hydroxy-5’-methylacetophenon and 4.6 ml (1 equiv.; 33.4 mmol) N,N-dimethylformamide-dimethylacetal (DMF·DMA) were heated under neat conditions to 55 °C for 19 h. The resulting deep red solution was evacuated to dryness to give 5.97 g of **e2b** (87%, 29.1 mmol). TLC (EtOAc/cyHex 1/1): 0.27; ^{1}H NMR (300 MHz, Chloroform-d) δ 13.69 (s, 1H), 7.88 (d, J = 12.2 Hz, 1H), 7.51 – 7.41 (m, 1H), 7.17 (dd, J = 8.4, 2.2 Hz, 1H), 6.84 (d, J = 8.4 Hz, 1H), 5.78 (d, J = 12.1 Hz, 1H), 3.20 (s, 3H), 2.99 (s, 3H), 2.30 (s, 3H). ^{1}H NMR (300 MHz, Acetonitrile-d3) δ 14.01 (s, 1H), 7.89 (d, *J* = 12.0 Hz, 1H), 7.66 (d, *J* = 2.0 Hz, 1H), 7.18 (dd, *J* = 8.5, 2.0 Hz, 1H), 6.73 (d, *J* = 8.5 Hz, 1H), 5.88 (d, *J* = 12.0 Hz, 1H), 3.18 (s, 3H), 2.98 (s, 3H), 2.28 (s, 3H). Other analytical data matches published literature.[5,6]

Synthesis of H**L2b**. 0.51 g (1 equiv., 2.47 mmol) of **e2b** was mixed with 0.143 mL (1.1 equiv., 2.72 mmol) methylhydrazine in H_2O and heated to refluxing temperatures for 7 h. After cooling down to rt and aqueous work-up with CH_2Cl_2 (3 x 20 mL), an orange-brown mixture of H**L2b** and H**L2b’** was obtained. After column chromatography (EtOAc/cyHex 1/1), 267.2 mg of H**L2b** were obtained as an orange-brown solid (48%, 1.31 mmol). TLC (EtOAc/cyHex 1/1): 0.55. Other analytical data matches published literature.[2]

Analytical data for H**L2b'**. TLC (EtOAc/cyHex 1/1): 0.23; ^{1}H NMR (300 MHz, Chloroform-d) δ 7.60 (t, *J* = 2.0 Hz, 1H), 7.17 – 7.11 (m, 1H), 7.00 (s, 1H), 6.91 (d, *J* = 8.3 Hz, 1H), 6.34 (d, *J* = 1.9 Hz, 1H), 5.02 (s, 1H), 3.79 (s, 3H), 2.32 (s, 3H).

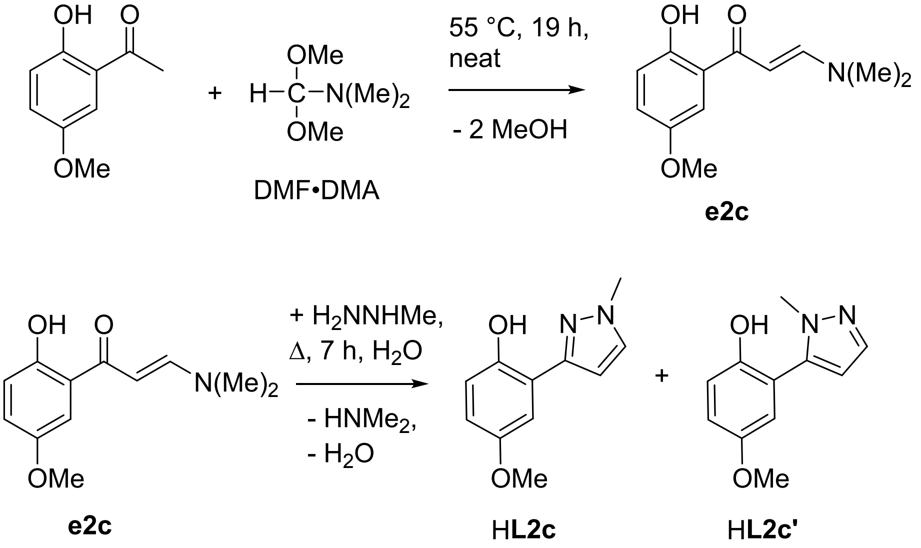

Scheme S4. Synthesis of H**L2c** via enamine **e2c**

Synthesis of **e2c**. 4.59 g (1 equiv., 27.6 mmol) 2′-Hydroxy-5′-methoxyacetophenone and 3.676 ml (1 equiv., 27.6 mmol) N,N-dimethylformamide-dimethylacetal (DMF·DMA) were heated under neat conditions to 55 °C for 19h. The resulting deep red solution was cooled to 8 °C to precipitate 5.56 g of **e2c** (91%, 25.1 mmol). TLC (EtOAc/cyHex 1/1): 0.19; ^{1}H NMR (300 MHz, Chloroform-d) δ 13.37 (s, 1H), 7.89 (d, *J* = 12.1 Hz, 1H), 7.19 (d, *J* = 3.0 Hz, 1H), 7.00 (dd, *J* = 9.0, 3.0 Hz, 1H), 6.88 (d, *J* = 9.0 Hz, 1H), 5.72 (d, *J* = 12.1 Hz, 1H), 3.80 (s, 3H), 3.20 (s, 3H), 2.98 (s, 3H). Other analytical data matches published literature.[5]

Synthesis of H**L2c**. 0.52 g (1 equiv., 2.34 mmol) of **e2c** was mixed with 0.135 mL (1.1 equiv., 2.57 mmol) methylhydrazine in H_2O and heated to refluxing temperatures for 7 h. After cooling down to rt and aqueous work-up with CH_2Cl_2 (3 x 20 mL), an orange-brown mixture of H**L2c** and H**L2c'** was obtained. After column chromatography (EtOAc/cyHex 1/1), 209.3 mg of H**L2c** were obtained as a yellow-brown solid (42%, 1.02 mmol). TLC (EtOAc/cyHex 1/1): H**L2c** = 0.45. Other analytical data matches published literature.[2]

Analytical data for H**L2c'**. TLC (EtOAc/cyHex 1/1): 0.16; ^{1}H NMR (300 MHz, Chloroform-d) δ 7.60 (d, *J* = 1.9 Hz, 1H), 6.97 – 6.88 (m, 2H), 6.74 (dd, *J* = 2.6, 0.8 Hz, 1H), 6.35 (d, *J* = 1.9 Hz, 1H), 4.91 (s, 1H), 3.80 (s, 3H), 3.79 (s, 3H).

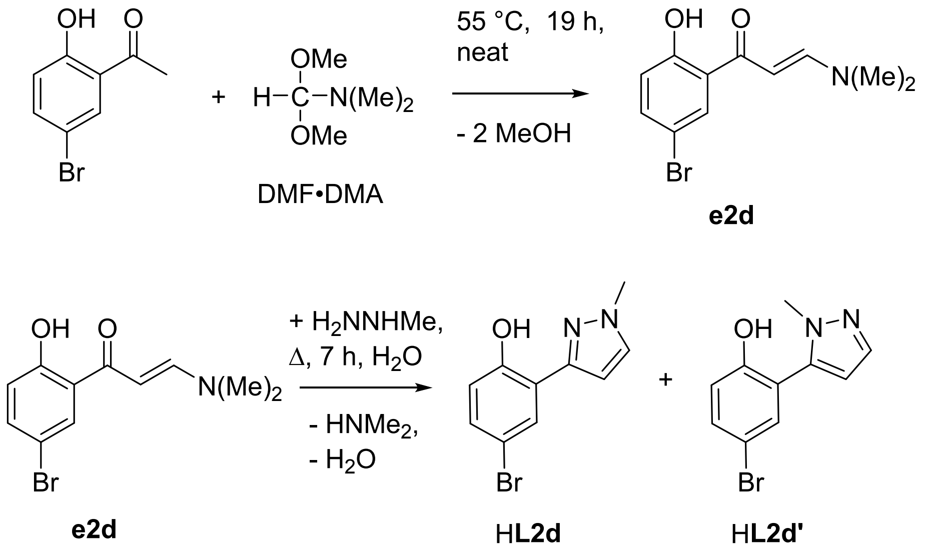

Scheme S5. Synthesis of H**L2d** via enamine **e2d**

Synthesis of **e2d**. 4.59 g (1 equiv., 21.3 mmol) 2′-Hydroxy-5′-bromoacetophenone and 2.84 ml (1 equiv., 21.3 mmol) N,N-dimethylformamide-dimethylacetal (DMF·DMA) were heated under neat conditions to 55 °C for 19 h. The resulting deep red solution was cooled to 8 °C to precipitate 4.91 g of **e2d** (91%, 19.4 mmol). TLC (EtOAc/cyHex 1/1): 0.29; ^{1}H NMR (300 MHz, Chloroform-d) δ 13.96 (s, 1H), 7.89 (d, *J* = 12.0 Hz, 1H), 7.76 (d, *J* = 2.4 Hz, 1H), 7.40 (dd, *J* = 8.8, 2.4 Hz, 1H), 6.82 (d, *J* = 8.8 Hz, 1H), 5.66 (d, *J* = 12.0 Hz, 1H), 3.20 (s, 3H), 2.99 (s, 3H). The data matches with published data.[5]

Synthesis of H**L2d**. 0.1 g (1 equiv., 0.37 mmol) **e2d** in 10 mL methanol and 0.021 ml (1.1 equiv., 0.41 mmol) methylhydrazine were mixed. The solution was heated to refluxing temperatures for 4 h. After cooling down to rt and aqueous work-up with CH_2Cl_2 (3 x 20 mL), an orange mixture of H**L2d** and H**L2d'** was obtained. After column chromatography (EtOAc/cyHex 1/1), 258 mg of H**L2d** were obtained as a yellow-brown solid (52%, 1.02 mmol). TLC (EtOAc/cyHex 1/1): H**L2d** = 0.41. ^{1}H NMR (300 MHz, Chloroform-*d*) δ 10.82 (s, 1H), 7.64 (d, *J* = 2.5 Hz, 1H), 7.41 (d, *J* = 2.4 Hz, 1H), 7.39 – 7.22 (m, 1H), 6.90 (d, *J* = 8.7 Hz, 1H), 6.58 (d, *J* = 2.4 Hz, 1H), 3.95 (s, 3H). The data matches with published data.[7]

Analytical data for H**L2d'**: TLC (EtOAc/cyHex 1/1): 0.20; ^{1}H NMR (300 MHz, Chloroform-*d*) δ 7.60 (d, *J* = 1.9 Hz, 1H), 7.44 (dd, *J* = 8.7, 1.9 Hz 1H), 7.33 (d, *J* = 2.4 Hz, 1H), 6.91 (d, *J* = 8.7 Hz, 1H), 6.36 (d, *J* = 1.9 Hz, 1H), 5.35 (s, 1H, OH), 3.79 (s, 3H).

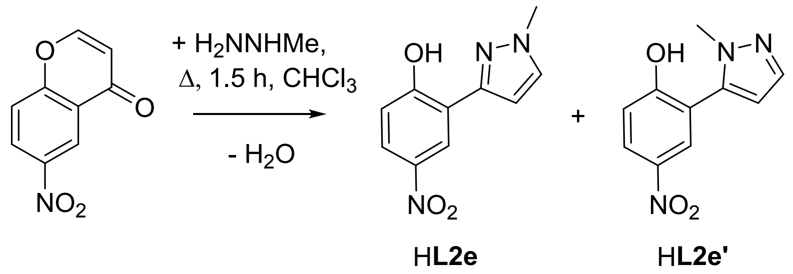

Scheme S6. Synthesis of **HL2e** from commercially available 6-nitrochromone.

The synthesis starting from commercially available 6-nitrochromone of ligand **HL2e** was previously published.[2]

Analytical data for **HL2e'**. TLC (EtOAc/cyHex 1/1): 0.16; ^{1}H NMR (300 MHz, Chloroform-d) δ 8.26 (app. d, *J* = 9.0 Hz, 1H), 8.18 (s, 1H), 7.65 (s, 1H), 7.12 (d, *J* = 9.0 Hz, 1H), 6.43 (app. s, 1H), 3.99 (s, 1H, OH), 3.82 (s, 3H).

Isolation of complex [ReO(OMe)(**L2e**)$_2$] (**6e'**)

A mixture of [$ReOCl_3(OPPh_3)(SMe_2)$] (**P1**) (350 mg, 0.54 mmol, 1 equiv.) and **HL2e** (249 mg, 1.13 mmol, 2.1 equiv.) and 2,6-lutidine (313 µl, 2.7 mmol, 5 equiv.) was heated to refluxing temperature in MeOH for 22 h. After cooling to room temperature, the green reaction solution was concentrated to remove most of the $OPPh_3$ and some lut·HCl by crystallization. NMR spectroscopy showed a mixture of app. 64% of **2e'** and 36% of **6e'** had formed. Purification of **6e'** was achieved by repeatedly washing the crude reaction mixture with small amounts of MeOH, as **6e'** was more soluble than **2e'**. ^{1}H NMR (300 MHz, Acetonitrile-d_3) δ 8.63 (d, *J* = 2.8 Hz, 1H), 8.42 (d, *J* = 2.9 Hz, 1H), 8.27 (dd, *J* = 9.0, 2.9 Hz, 1H), 7.97 (d, *J* = 2.8 Hz, 1H), 7.69 (dd, *J* = 9.1, 2.8 Hz, 1H), 7.48 (d, *J* = 2.7 Hz, 1H), 7.41 (d, *J* = 9.1 Hz, 1H), 7.05 (d, *J* = 2.7 Hz, 1H), 6.87 (d, *J* = 2.8 Hz, 1H), 5.99 (d, *J* = 9.1 Hz, 1H), 4.85 (s, 3H), 4.30 (s, 3H), 3.49 (s, 3H, -OCH_3). ATR-IR (cm^{-1}): 3133 (w), 2850 (w), 1574 (m) 1503 (s, C=N), 1317 (s), 1129 (m), 963 (Re=O, m), 879 (m), 735 (s), 424 (m); EI-MS (*m/z*): 670 [M$^+$], 639 [M$^+$ - OMe]; Elemental analysis calculated for $C_{21}H_{19}N_6O_8Re$ (670.08): C 37.67, H 2.86, N 12.55; found: C 37.62, H 2.73, N 12.35.

NMR spectra

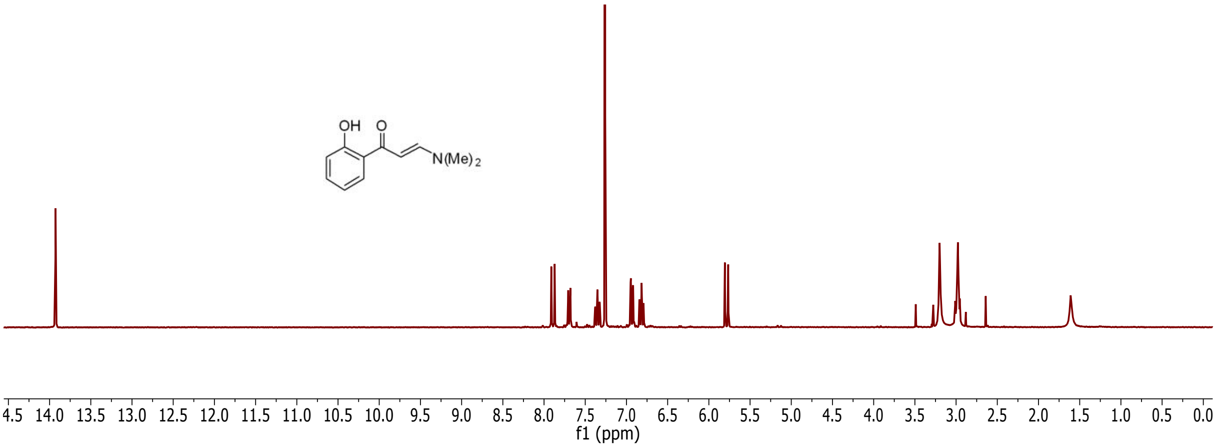

Figure S1. ^{1}H NMR spectrum of **e2a** ($CDCl_3$).

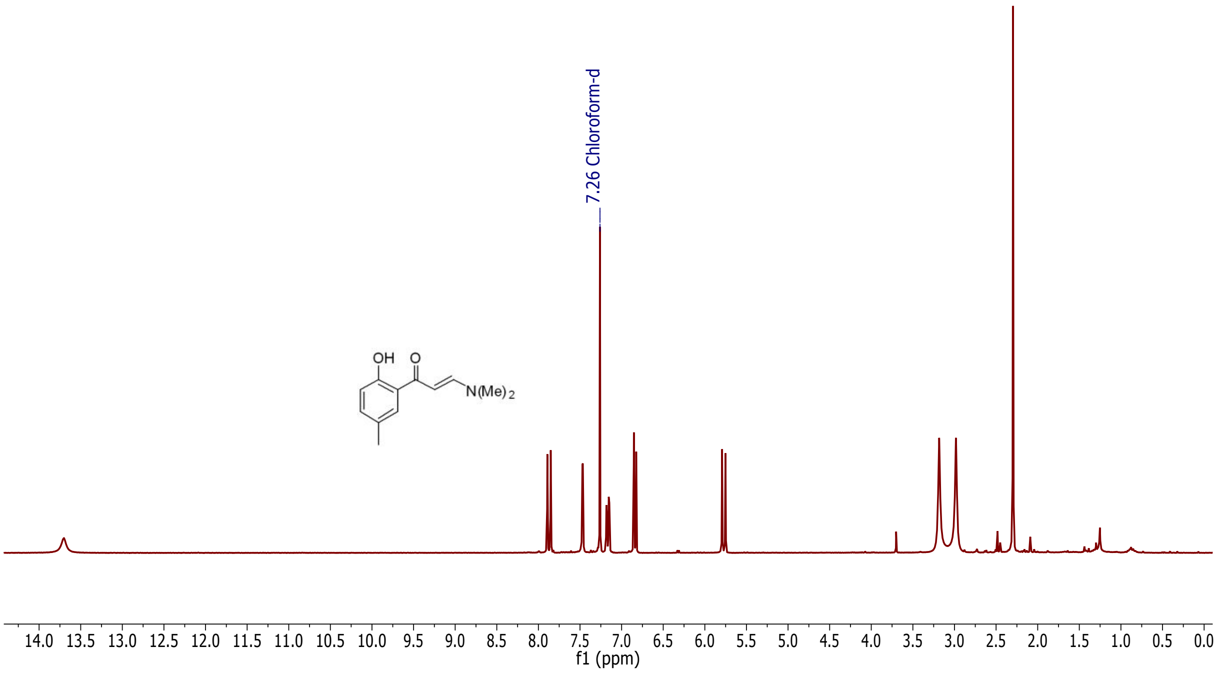

Figure S2. ^{1}H NMR spectrum of **e2b** ($CDCl_3$).

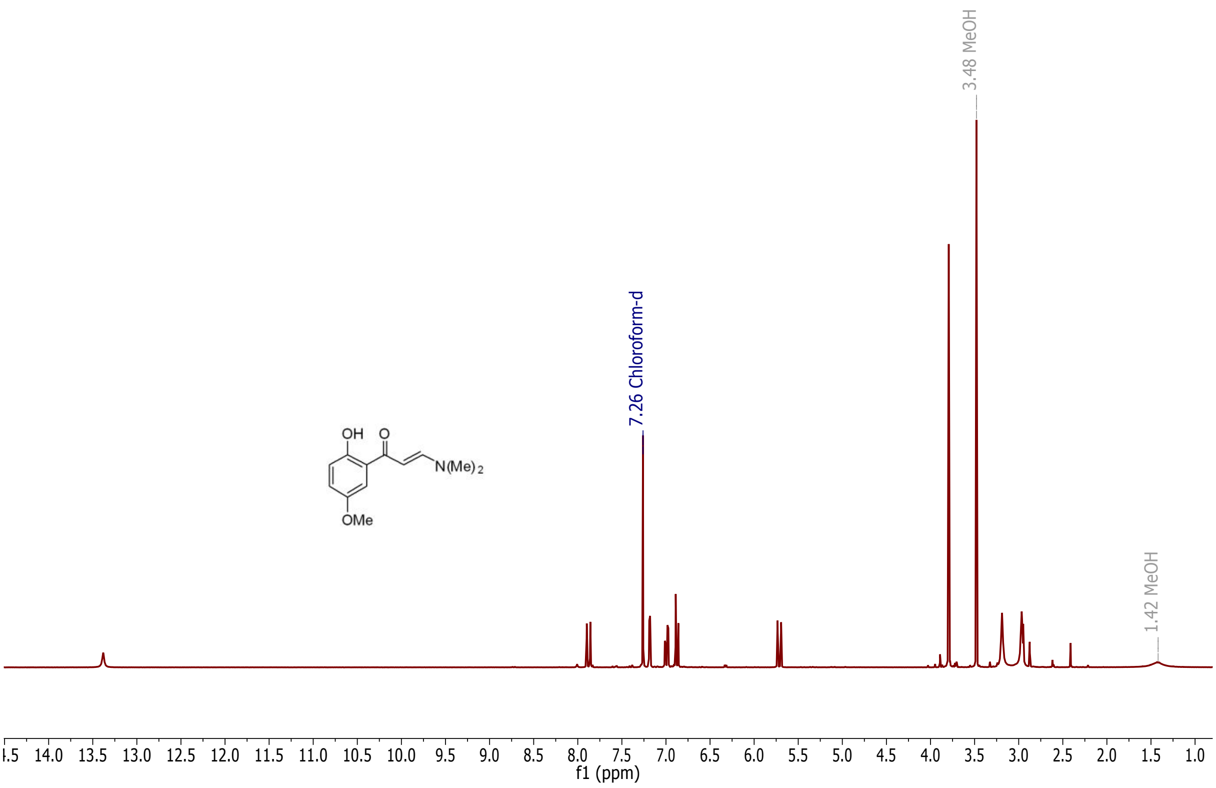

Figure S3. ^{1}H NMR spectrum of **e2c** ($CDCl_3$).

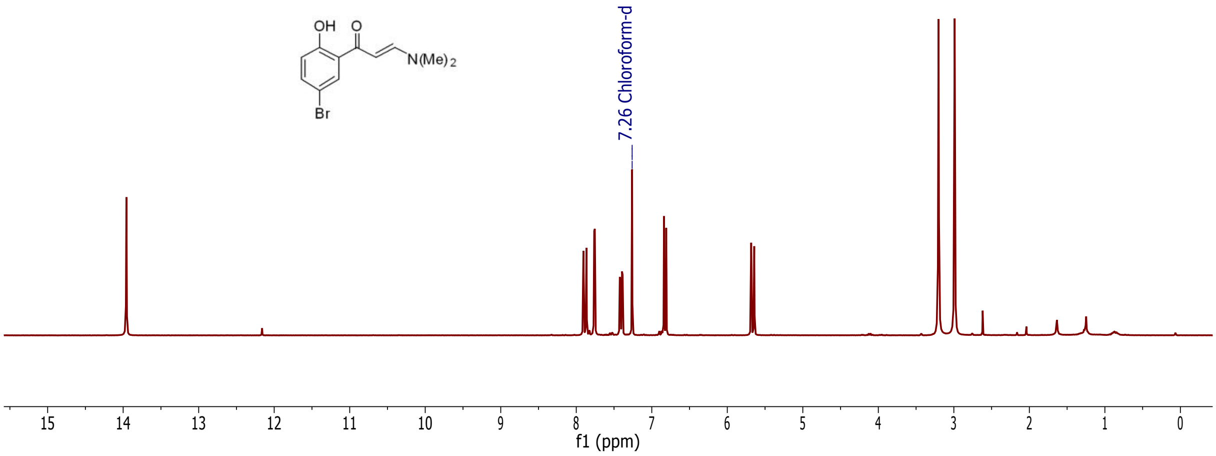

Figure S4. ^{1}H NMR spectrum of **e2d** ($CDCl_3$).

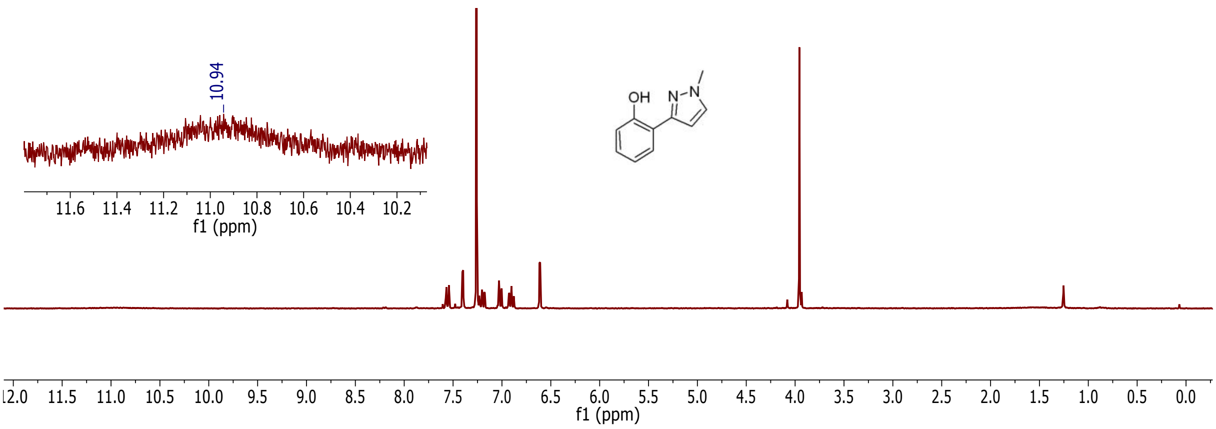

Figure S5. ^{1}H NMR spectrum of **HL2a** with zoom-in for -OH peak ($CDCl_3$).

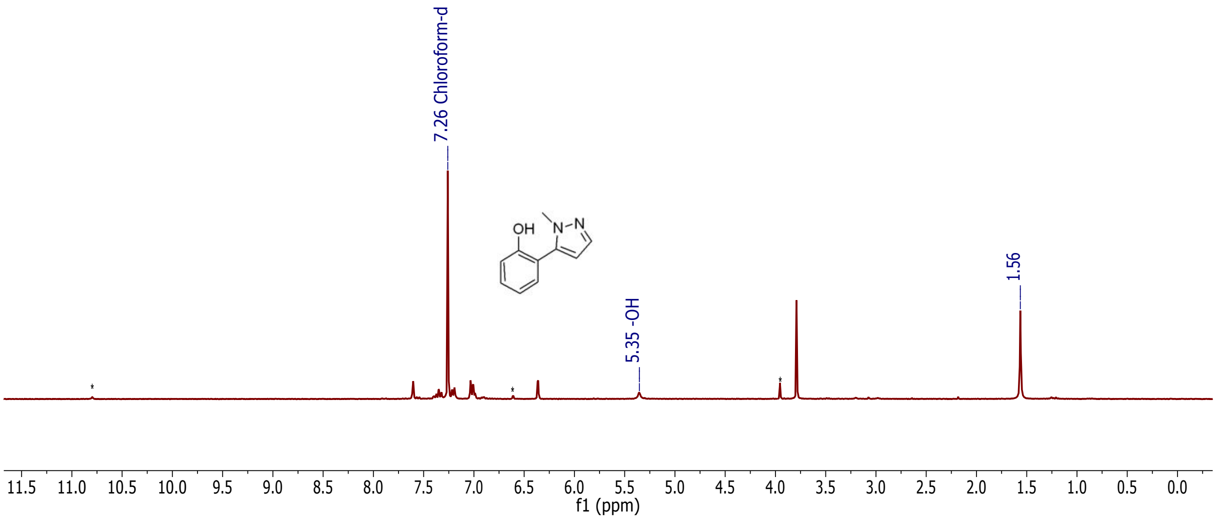

Figure S6. ^{1}H NMR spectrum of a 86/14% mixture of H**L2a'**/H**L2a** (non-overlapping peaks of H**L2a** are labelled with an asterisk ($CDCl_3$). The diagnostic, up-field shifted -OH peak of H**L2a'** (5.35 ppm) is labelled.

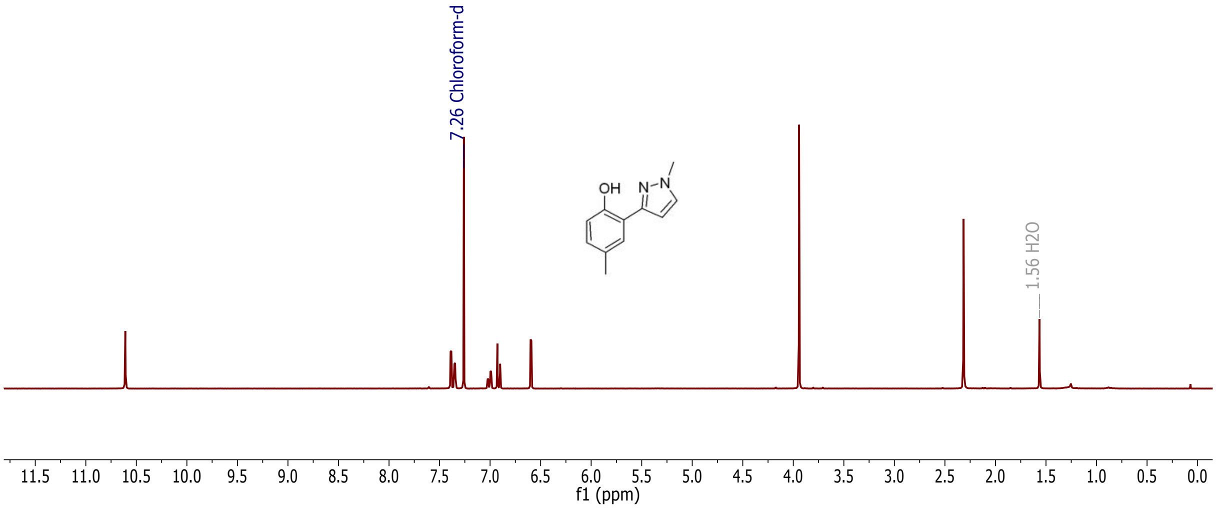

Figure S7. ^{1}H NMR spectrum of **HL2b** ($CDCl_3$).

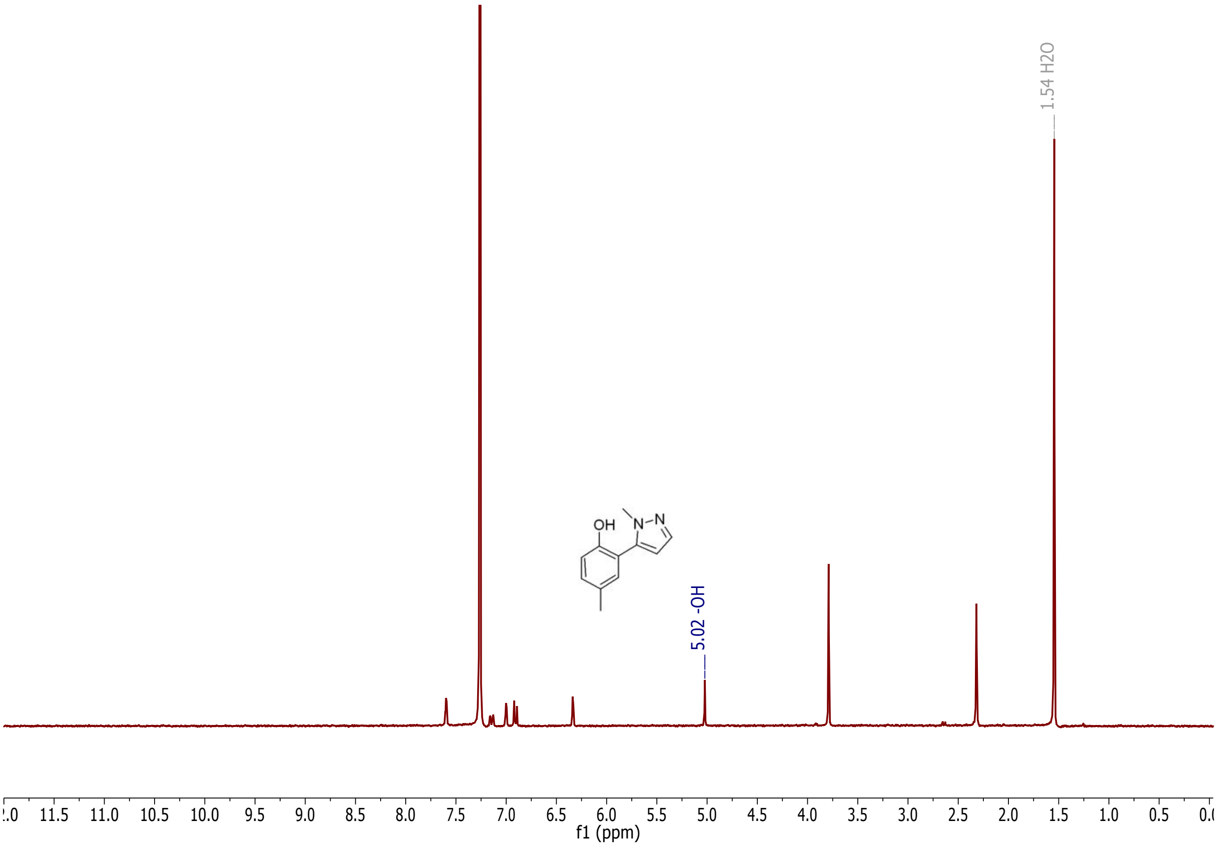

Figure S8. ¹H NMR spectrum of H**L2b'**. The diagnostic, up-field shifted -OH peak of H**L2b'** (5.02 ppm) is labelled.

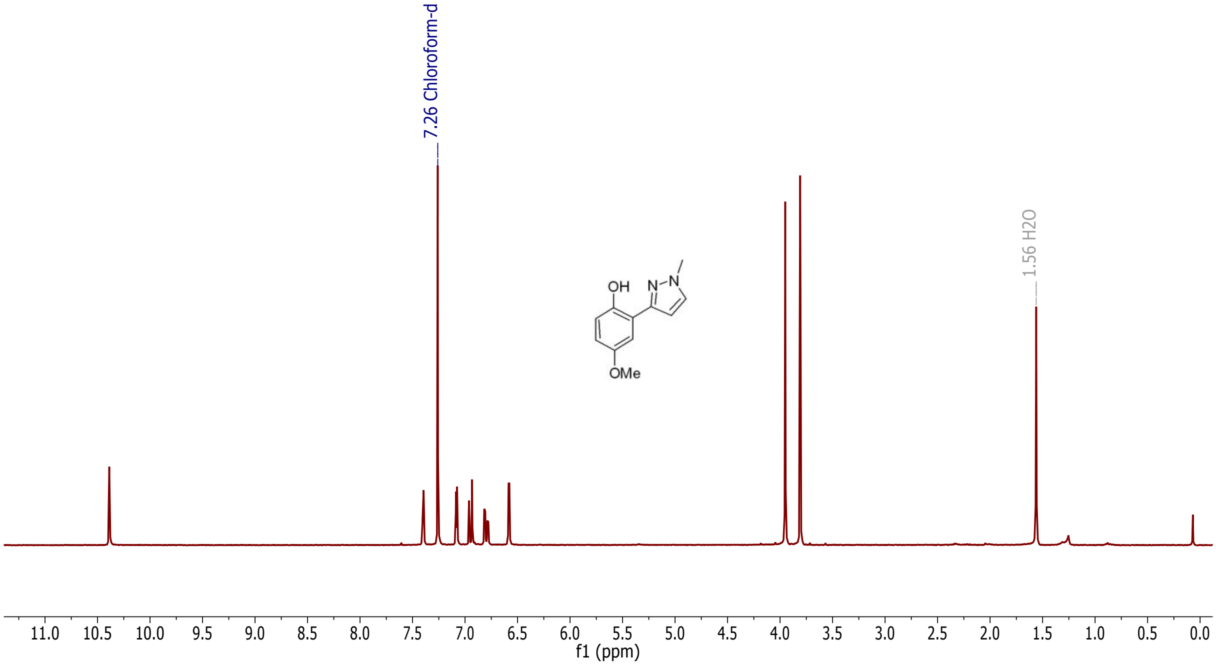

Figure S9. ¹H NMR spectrum of **HL2c** ($CDCl_3$).

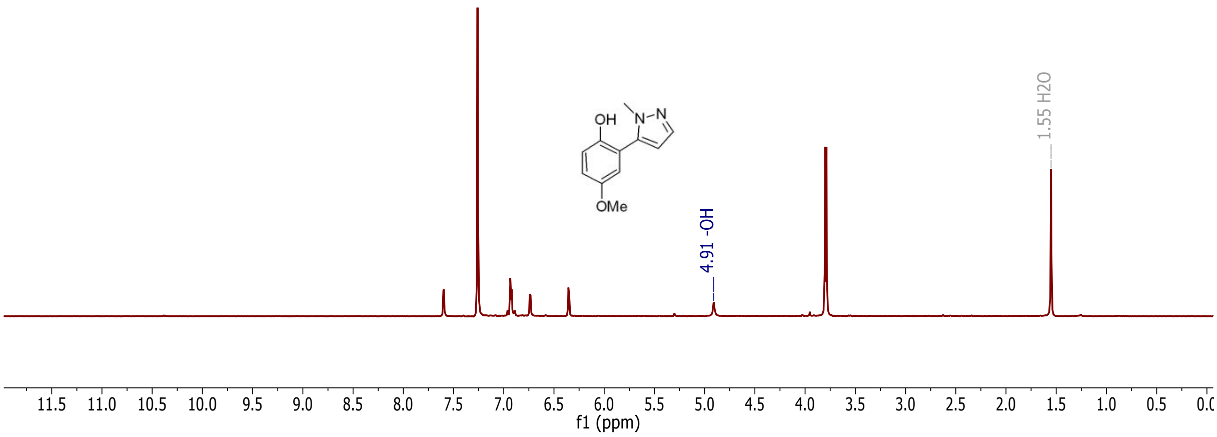

Figure S10. ^{1}H NMR spectrum of H**L2c'**. The diagnostic, up-field shifted -OH peak of H**L2c'** (4.91 ppm) is labelled.

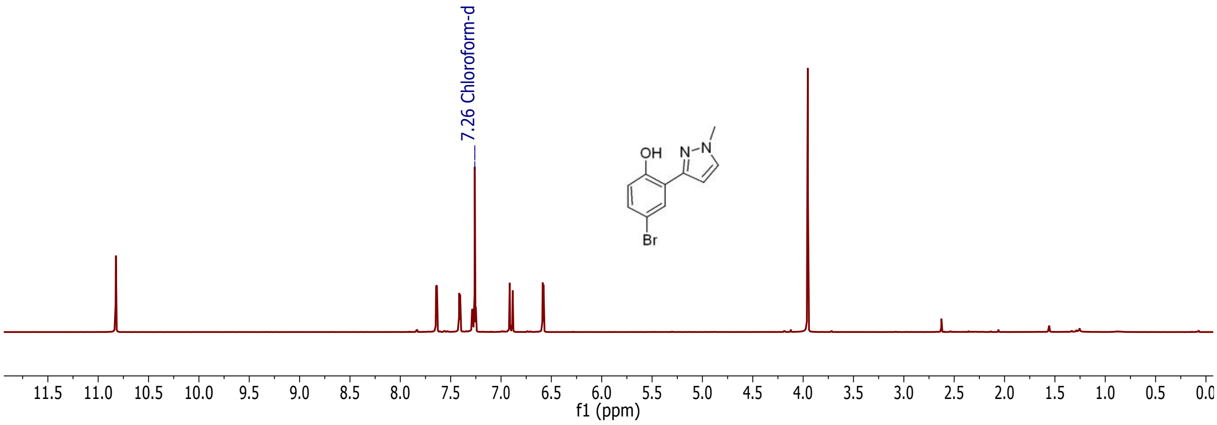

Figure S11. ^{1}H NMR spectrum of **HL2d** ($CDCl_3$).

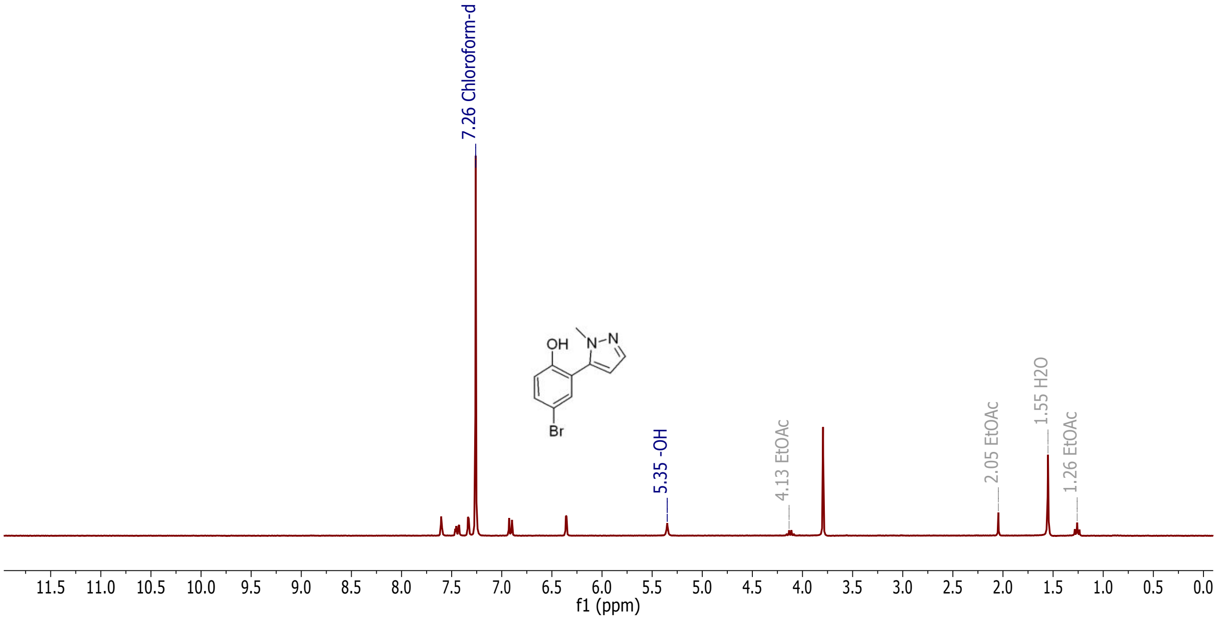

Figure S12. ^{1}H NMR spectrum of HL2d’. The diagnostic, up-field shifted -OH peak of HL2d’ (5.35 ppm) is labelled.

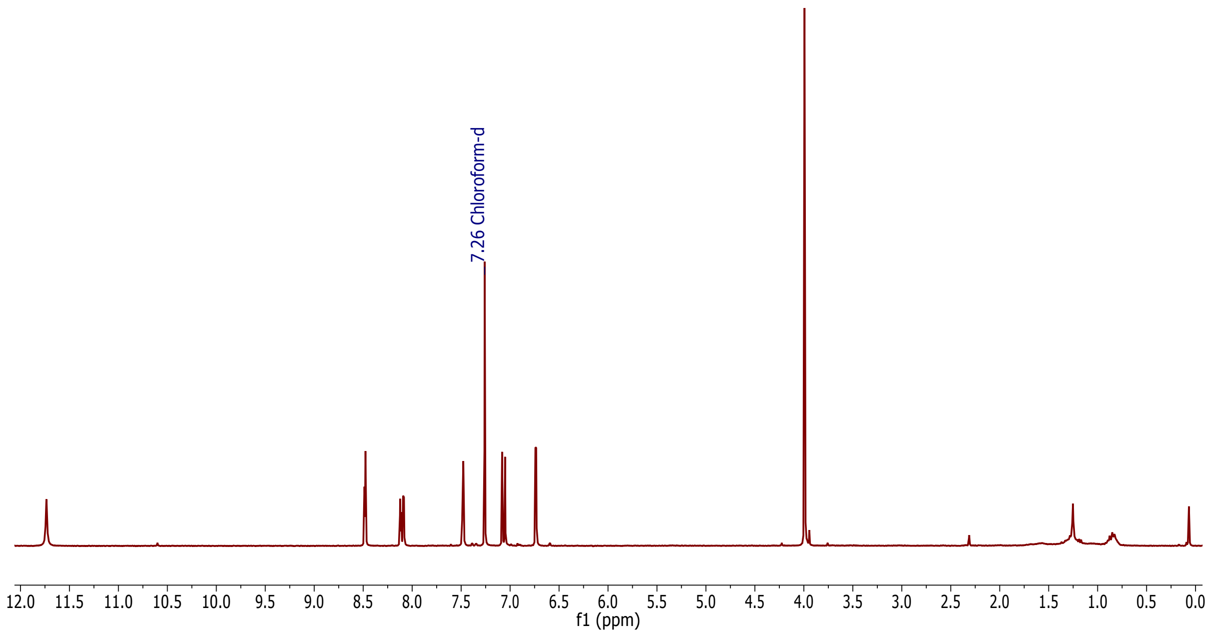

Figure S13. ^{1}H NMR spectrum of **HL2e** ($CDCl_3$).

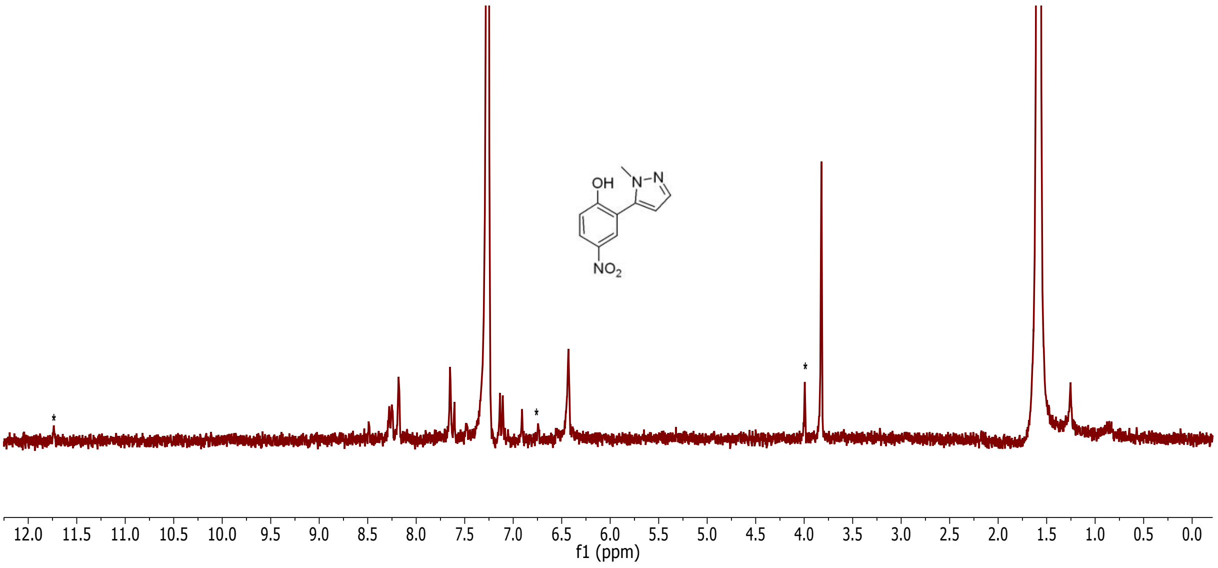

Figure S14. ^{1}H NMR spectrum of a 83/17% mixture of HL2e’/HL2e (non-overlapping peaks of HL2e are labelled with an asterisk ($CDCl_3$).

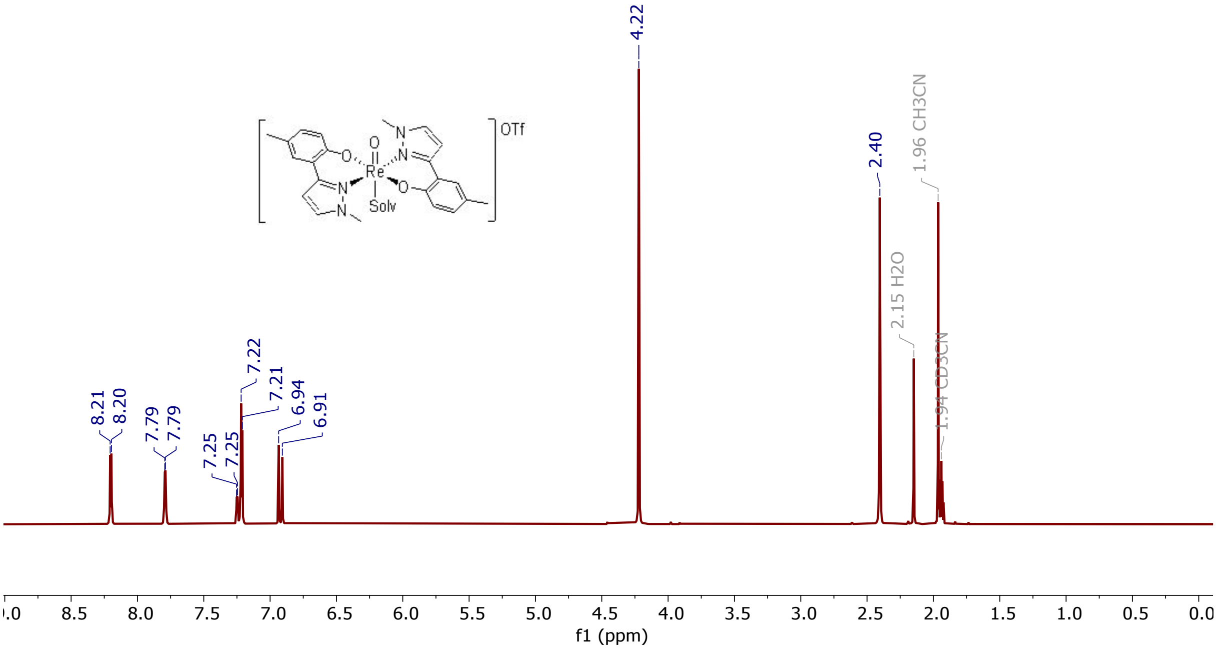

Figure S15. ^{1}H NMR spectrum of **4b** (CD_3CN).

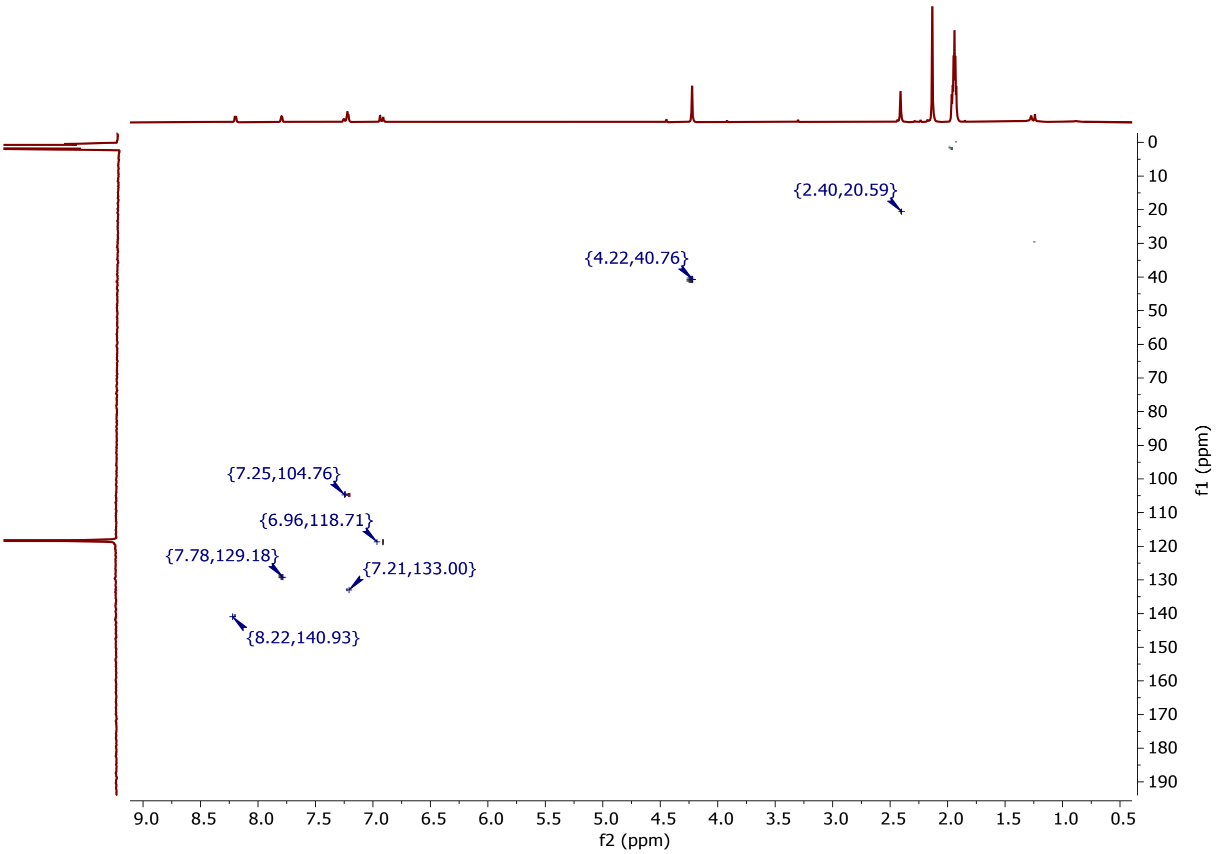

Figure S16. HSQC spectrum of **4b** (CD_3CN).

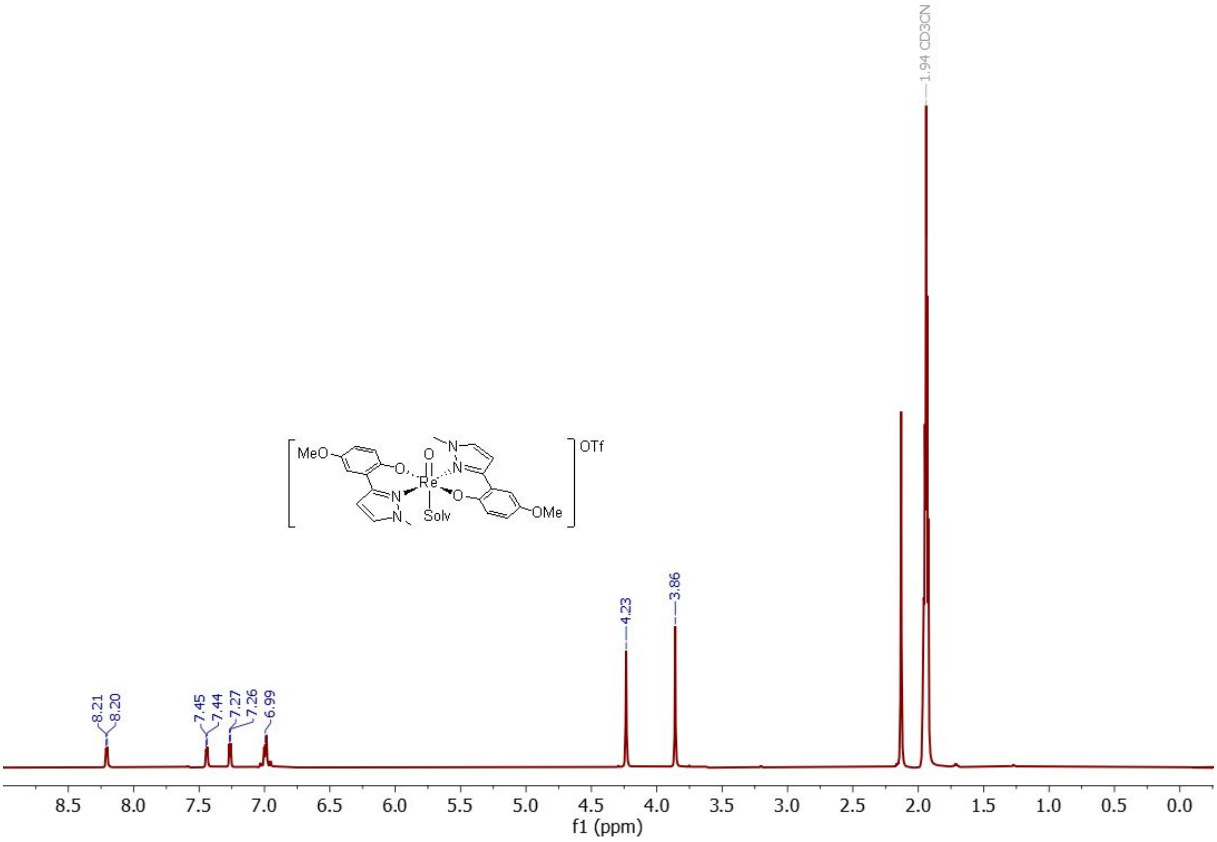

Figure S17. ^{1}H NMR spectrum of **4c** (CD_3CN).

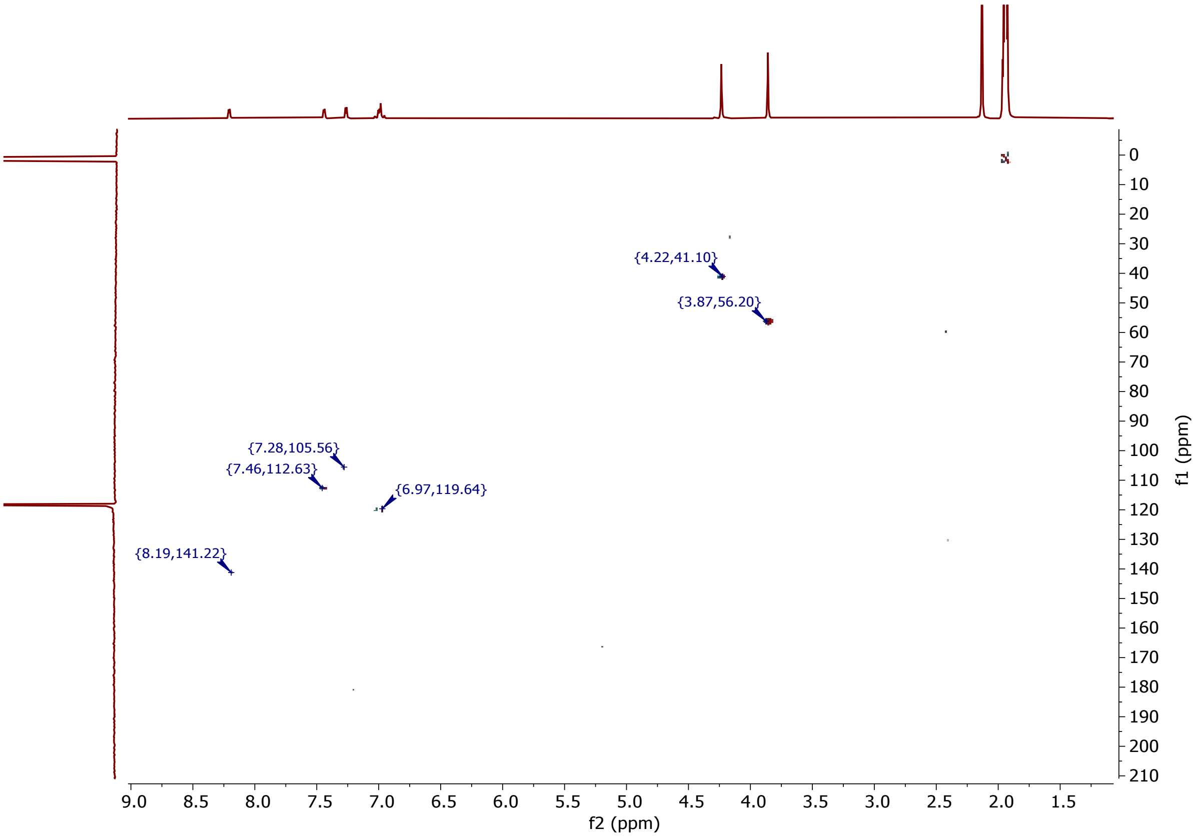

Figure S18. HSQC spectrum of **4c** (CD_3CN).

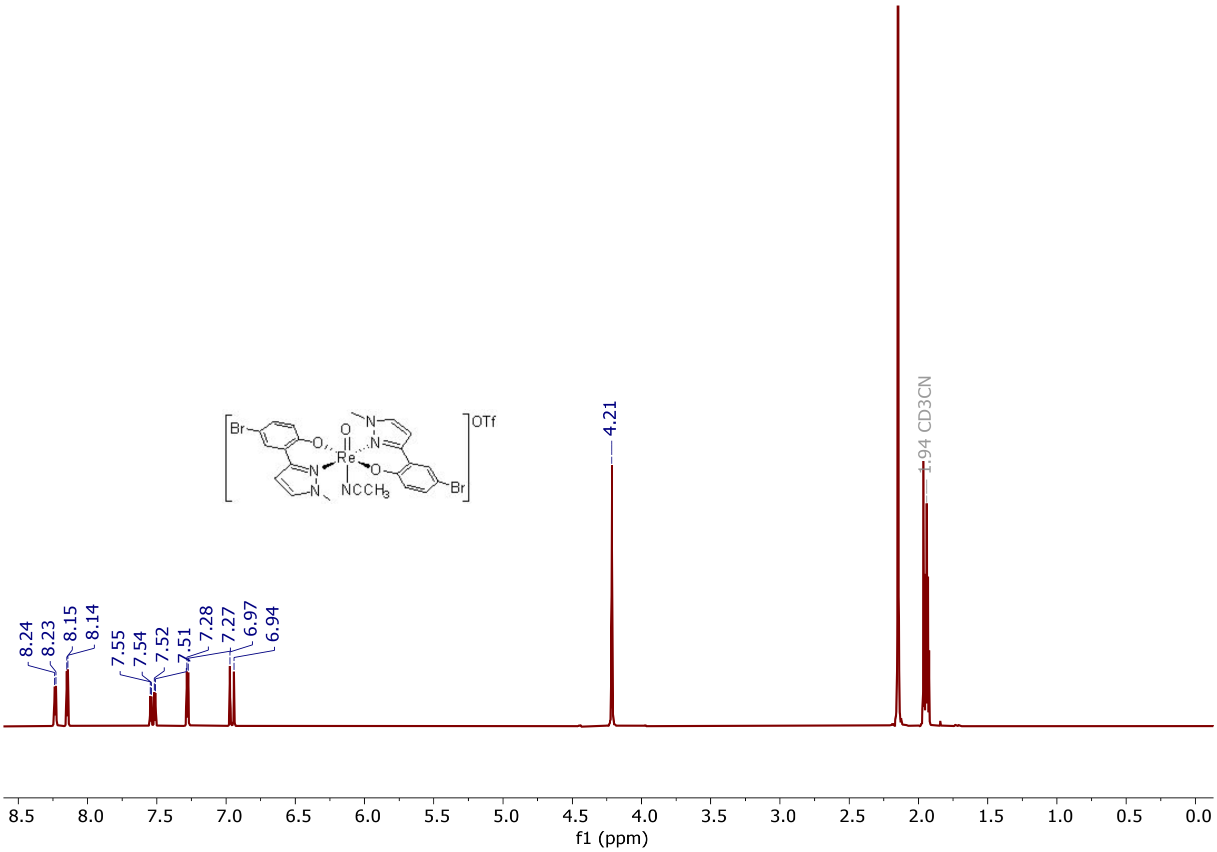

Figure S19. ¹H NMR spectrum of **4d** (CD_3CN).

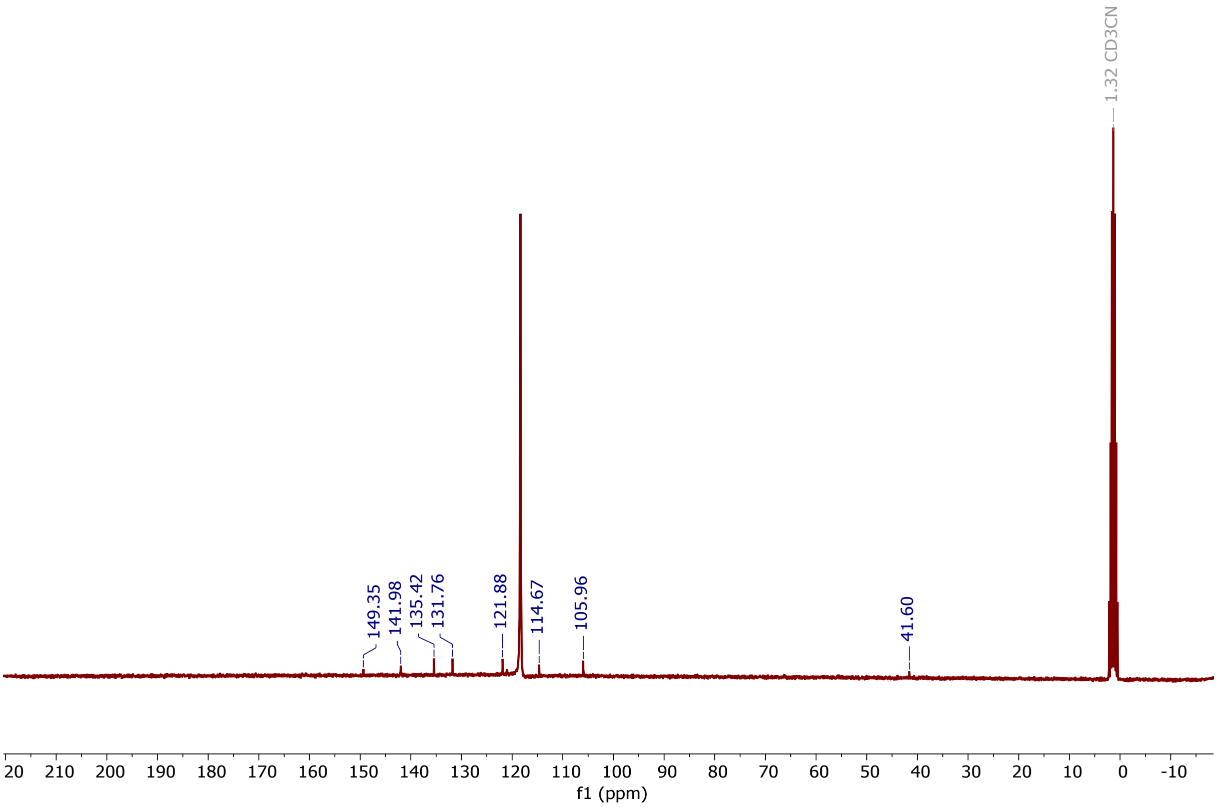

Figure S20. ^{13}C NMR spectrum of **4d** (CD_3CN).

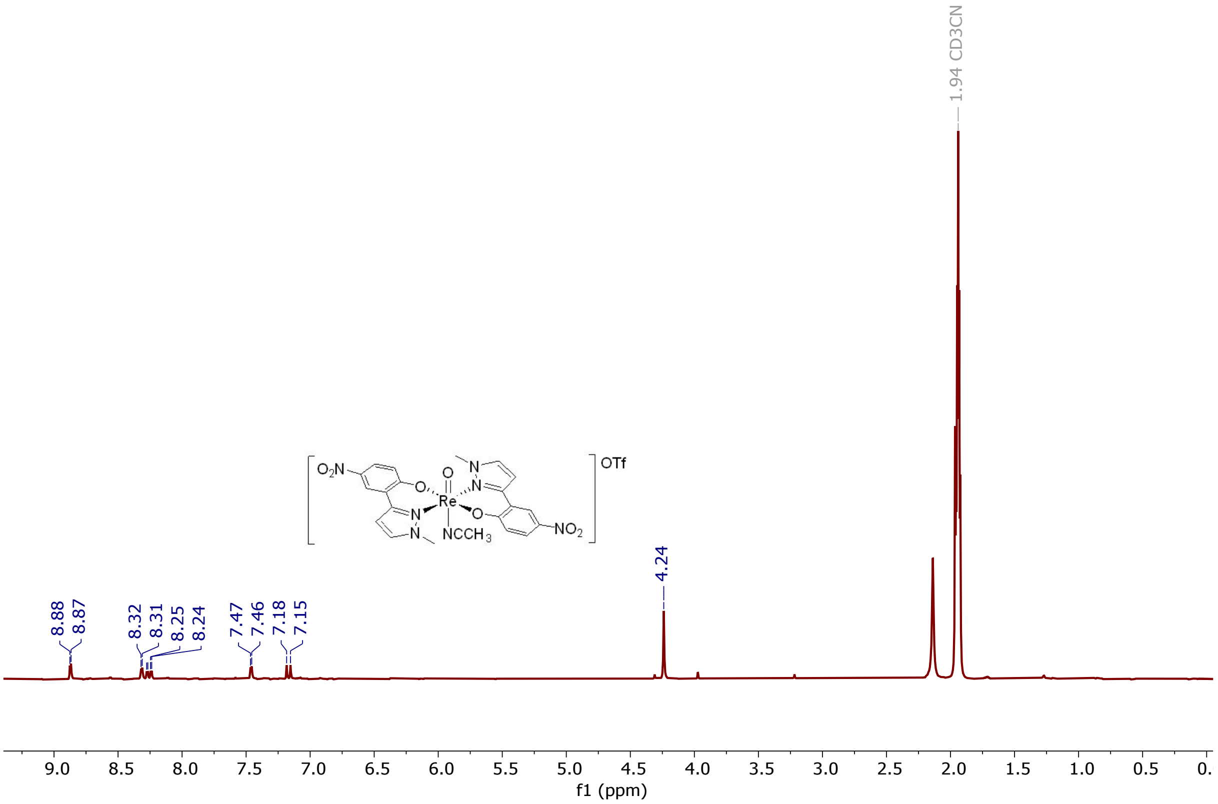

Figure S21. ^{1}H NMR spectrum of **4e** (CD_3CN).

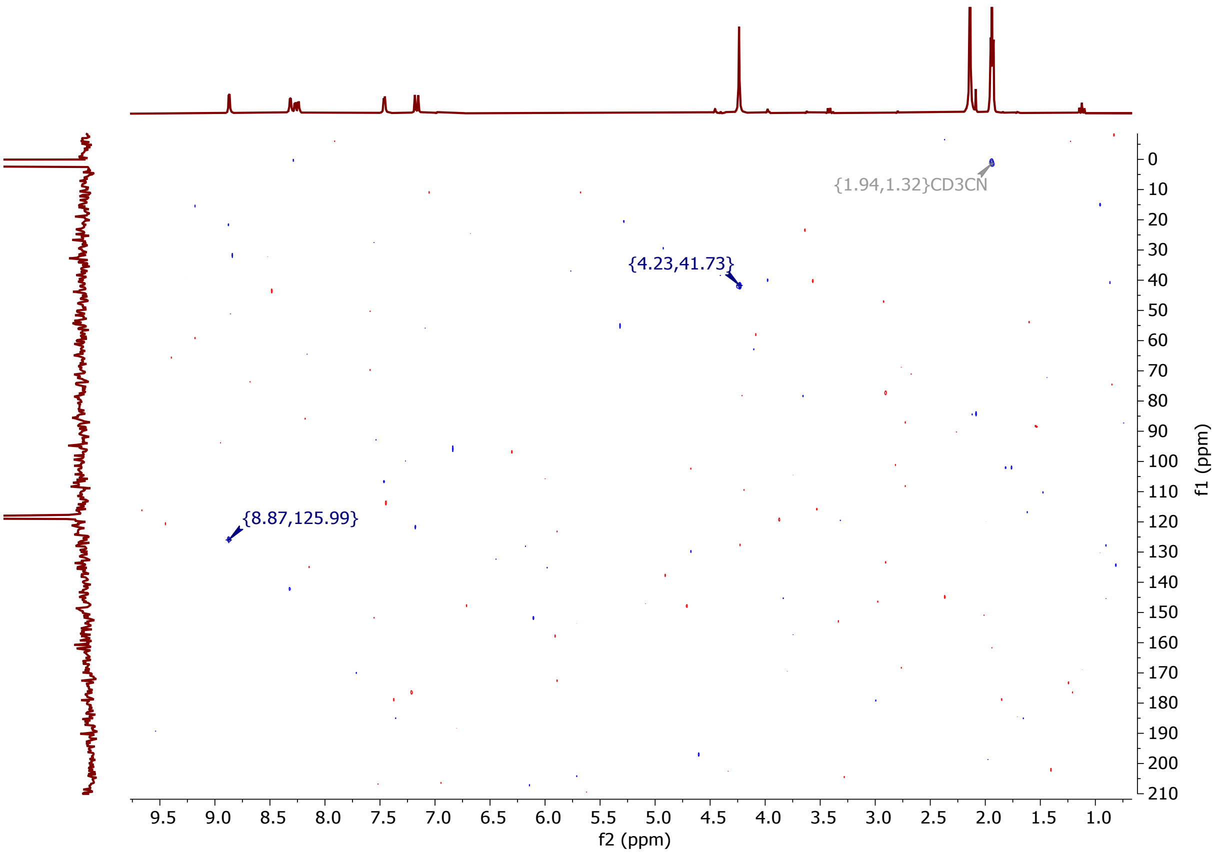

Figure S22. HSQC spectrum of **4e** (CD_3CN) (solubility was very low).

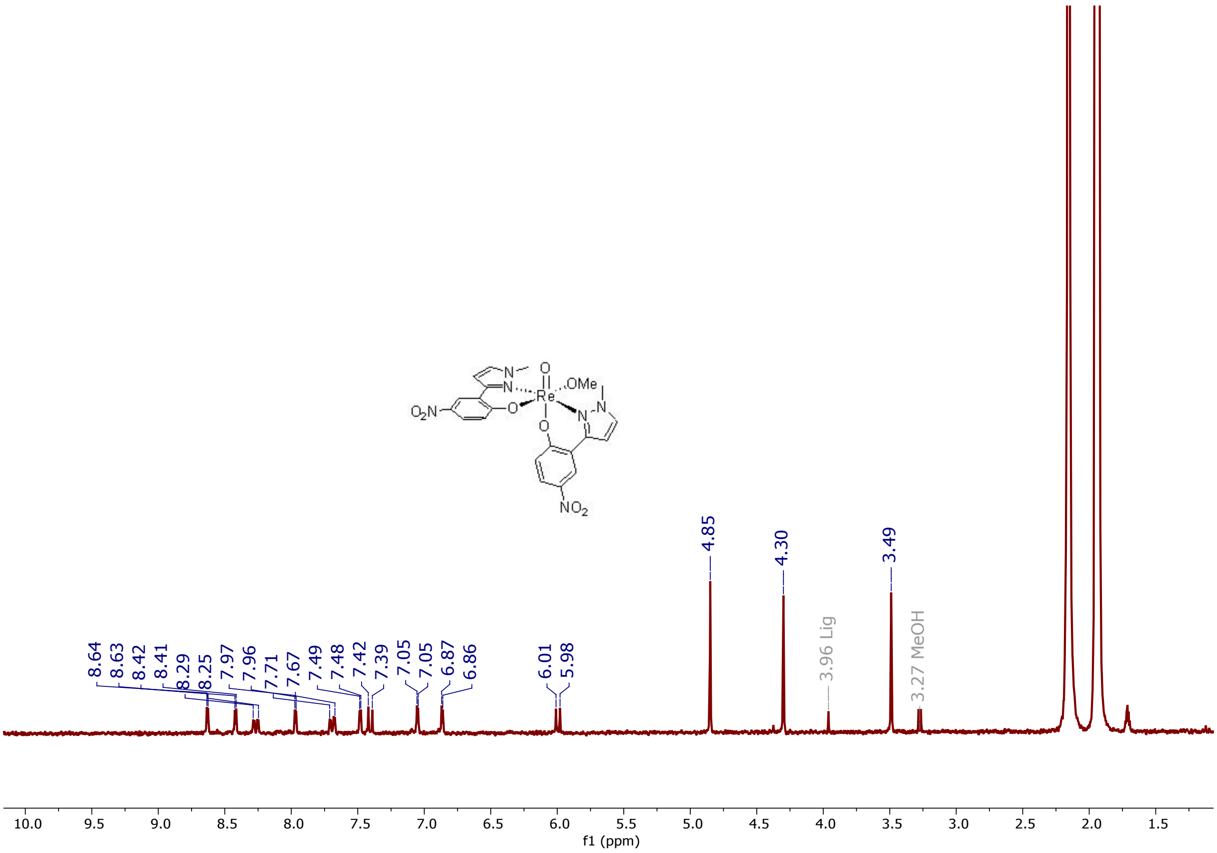

Figure S23. ^{1}H NMR spectrum of **6e'** (CD_3CN).

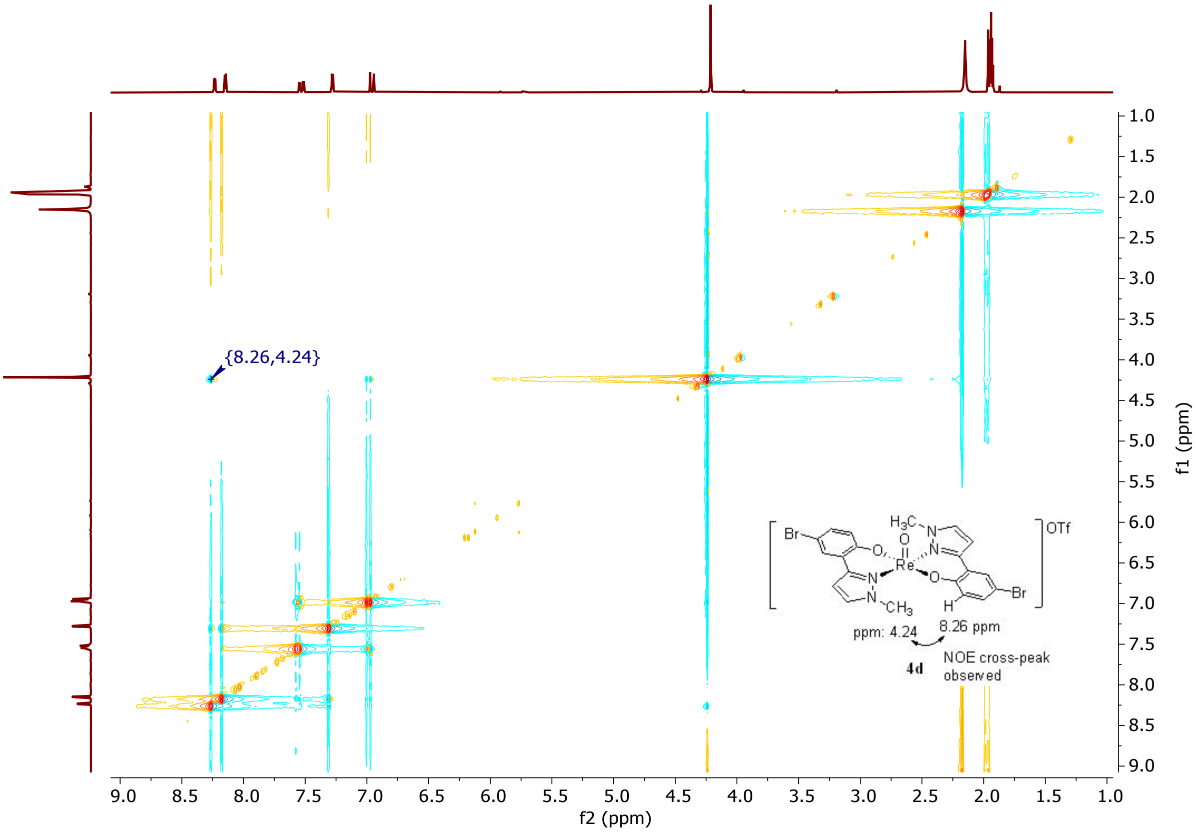

Figure S24. 2D-NOESY spectrum of **4d**, showing an NOE cross peak, proving that the cationic triflate complex remains in N,N-*trans* conformation in solution.

Table S1. Overview of reaction outcomes for the attempted synthesis of N,N-*trans* **2a**

	base	equiv. base	solvent	t [h][a]	result
1	pyridine	5	MeOH	22	**2a'**; potential formation of [ReOCl$_2$(**L2a**)(py)]
2	pyridine	5	CH_3CN	22	decomp.; reaction soln. turns brown
3	lutidine	5	MeOH	22	**2a'**; potential formation of [ReO(OMe)(**L2a**)$_2$]
4	lutidine	5	CH_3CN	19	only **2a'**
5	BDMEP	5	MeOH	20	only **2a'**
6	BDMEP	5	CH_3CN	20	only **2a'**

[a] heating to refluxing temp.

Table S2. Overview of reaction outcomes for the attempted synthesis of N,N-*trans* **2b**

	base	equiv. base	solvent	t [h][a]	result
1	pyridine	5	MeOH	22	potential formation of **2b**, but could not be isolated
2	pyridine	5	CH_3CN	22	decomp.; reaction soln. turns brown
3	lutidine	5	MeOH	22	potential formation of **2b**, but could not be isolated
4	lutidine	5	CH_3CN	22	only **2b'**
5	lutidine	10	MeOH	22	potential formation of **2b**, but could not be isolated
6	lutidine	3	MeOH	22	potential formation of **2b**, but could not be isolated
7	BDMEP	5	MeOH	20	only **2b'**
8	BDMEP	5	CH_3CN	20	only **2b'**

[a] heating to refluxing temp.

Table S3. Overview of reaction outcomes for the attempted synthesis of N,N-*trans* **1c**

	base	equiv. base	solvent	t [h][a]	result
1	pyridine	5	MeOH	22	only **2c'**
2	pyridine	5	CH_3CN	22	decomp.; reaction soln. turns brown
3	lutidine	5	MeOH	22	only **2c'**
4	lutidine	5	CH_3CN	19	only **2c'**
5	BDMEP	5	MeOH	20	only **2c'**
6	BDMEP	5	CH_3CN	20	only **2c'**

[a] heating to refluxing temp.

Table S4. Overview of reaction outcomes for the attempted synthesis of N,N-*trans* **2d**

	base	equiv. base	solvent	t [h][a]	result
1	pyridine	5	MeOH	22	**2a'**; potential formation of [$ReOCl_2$(**L2d**)(py)]
2	pyridine	5	CH_3CN	22	decomp.; reaction soln. turns brown
3	lutidine	5	MeOH	22	formation of **2d'** and **6d'**
4	lutidine	5	CH_3CN	19	only **2d'**
5	BDMEP	5	MeOH	20	only **2d'**
6	BDMEP	5	CH_3CN	20	only **2d'**

[a] heating to refluxing temp.

Table S5. Overview of reaction outcomes for the attempted synthesis of N,N-*trans* **2e**

	base	equiv. base	solvent	t [h][a]	result
1	pyridine	5	MeOH	22	formation of **2e'** and **5e**
2	pyridine	5	CH_3CN	22	decomp.; reaction soln. turns brown
3	lutidine	5	MeOH	22	formation of **2e'** and **6e'**
4	lutidine	5	CH_3CN	19	only **2d'**

**5**	BDMEP	5	MeOH	20	only **2d’**
**6**	BDMEP	5	CH_3CN	20	only **2d’**

[a] heating to refluxing temp.

Details on single-crystal X-ray crystallography

Crystal Structure Determination of 2b'. All the measurements were performed using monochromatized Mo K_a radiation at 100K: $C_{22}H_{22}ClN_4O_3Re$, M_r 612.08, monoclinic, space group C 2/c, a = 26.2006(12)Å, b = 12.9126(6)Å, c = 14.6652(6)Å, b = 121.9342(18)°, V = 4210.6(3)Å^3, Z = 8, d_{calc} = 1.931g cm^{-3}, m = 5.932mm^{-1}. A total of 85717 reflections were collected (Q_{max} = 30.0°), from which 6146 were unique (R_{int} = 0.0447), with 5476 having I > 2s(I). The structure was solved by direct methods (SHELXS-97)[8] and refined by full-matrix least-squares techniques against F^2 (SHELXL-2014/6)[9]. The non-hydrogen atoms were refined with anisotropic displacement parameters without any constraints. The H atoms of the aromatic rings were put at the external bisectors of the C–C–X angles at C–H distances of 0.95Å and common isotropic displacement parameters were refined for the H atoms of the same ring. The H atoms of the methyl groups were refined with common isotropic displacement parameters for the H atoms of the same group and idealized geometries with tetrahedral angles, enabling rotations around the C–C bonds, and C–H distances of 0.98Å. The largest peaks in a final difference Fourier map (0.74 - 1.40eÅ^{-3}) were in the vicinity (0.72 - 0.91Å) of the Re atom. For 292 parameters final R indices of R1 = 0.0182 and wR^2 = 0.0453 (GOF = 1.045) were obtained.

Table S6. Crystal data and structure refinement for **2b'**.

Crystal data	
Identification code	JSB36B
Empirical formula	$C_{22}H_{22}ClN_4O_3Re$
Formula weight	612.08
Crystal description	block, green
Crystal size	0.20 x 0.14 x 0.06mm
Crystal system, space group	monoclinic, C 2/c
Unit cell dimensions: a	26.2006(12)Å
b	12.9126(6)Å
c	14.6652(6)Å
β	121.9342(18)°
Volume	4210.6(3)Å^3
Z	8
Calculated density	1.931Mg/m^3
F(000)	2384
Linear absorption coefficient μ	5.932mm^{-1}
Absorption correction	semi-empirical from equivalents
Max. and min. transmission	0.746 and 0.497
Unit cell determination	2.82° < Θ < 29.97°
	9996 reflections used at 100K
Data collection	
Temperature	100K
Diffractometer	Bruker APEX-II CCD

Radiation source	Incoatec microfocus sealed tube
Radiation and wavelength	MoK_{α}, 0.71073Å
Monochromator	multilayer monochromator
Scan type	ϕ and ω scans
Θ range for data collection	2.10 to 30.00°
Reflections collected / unique	85717 / 6146
Significant unique reflections	5476 with $I > 2\sigma(I)$
R(int), R(sigma)	0.0447, 0.0237
Completeness to Θ = 30.0°	100.0%
Refinement	
Refinement method	Full-matrix least-squares on F^2
Data / parameters / restraints	6146 / 292 / 0
Goodness-of-fit on F^2	1.045
Final R indices [$I > 2\sigma(I)$]	R1 = 0.0182, wR2 = 0.0441
R indices (all data)	R1 = 0.0227, wR2 = 0.0453
Extinction expression	none
Weighting scheme	$w = 1/[\sigma^2(F_o^2)+(aP)^2+bP]$ where $P = (F_o^2+2F_c^2)/3$
Weighting scheme parameters a, b	0.0221, 3.9663
Largest Δ/σ in last cycle	0.001
Largest difference peak and hole	1.401 and -0.861e/Å^3
Structure Solution Program	SHELXS-97[8]
Structure Refinement Program	SHELXL-2014/6[9]
CCDC number	2468748

Table S7. Selected bond lengths [Å] and angles [°] for **2b'**

Re1-O1	1.6943(15)
Re1-O2	1.9836(14)
Re1-O4	1.9725(14)
Re1-N12	2.1221(16)
Re1-N32	2.1340(17)
Re1-Cl1	2.3617(5)
O2-C21	1.338(2)
O4-C41	1.342(2)

O1-Re1-O2	163.32(6)
O4-Re1-N12	163.51(6)
N32-Re1-Cl1	171.05(5)
C13-N12-N11	105.98(16)
C13-N12-Re1	127.64(13)
N11-N12-Re1	125.00(13)
C21-O2-Re1	128.50(12)
C33-N32-N31	106.70(16)
C33-N32-Re1	124.35(13)
N31-N32-Re1	128.02(13)
C41-O4-Re1	126.44(12)

Crystal Structure Determination of 4b. All the measurements were performed using monochromatized Mo K_{α} radiation at 100K: $C_{24}H_{25}N_5O_3Re^{+}CF_3O_3S^{-}$, M_r 766.76, monoclinic, space group P 2_1/n, a = 12.3990(6)Å, b = 14.6632(7)Å, c = 14.8763(8)Å, β = 91.382(3)°, V = 2703.9(2)Å^3, Z = 4, d_{calc} = 1.884g cm^{-3}, μ = 4.643mm^{-1}. A total of 65905 reflections were collected (Θ_{max} = 30.0°), from which 7832 were unique (R_{int} = 0.1122), with 5681 having I > 2σ(I). The structure was solved by direct methods (SHELXS-97)[8] and refined by full-matrix least-squares techniques against F^2 (SHELXL-2014/6)[9]. The non-hydrogen atoms were refined with anisotropic displacement parameters without any constraints. The H atoms of the aromatic rings were put at the external bisectors of the X–C–C angles at C–H distances of 0.95Å and common isotropic displacement parameters were refined for the H atoms of the same ring. The H atoms of the methyl groups were refined with common isotropic displacement parameters for the H atoms of the same group and idealized geometries with tetrahedral angles, enabling rotations around the X–C bonds, and C–H distances of 0.98Å. For 384 parameters final *R* indices of R1 = 0.0435 and wR^2 = 0.0967 (GOF = 1.037) were obtained. The largest peak in a difference Fourier map was 1.842eÅ^{-3}.

Table S8. Crystal data and structure refinement for **4b**.

Crystal data	
Identification code	JSBM58
Empirical formula	$C_{24}H_{25}N_5O_3Re^{+}CF_3O_3S^{-}$
Formula weight	766.76
Crystal description	block, orange
Crystal size	0.16 x 0.09 x 0.07mm
Crystal system, space group	monoclinic, P 2_1/n
Unit cell dimensions: a	12.3990(6)Å
b	14.6632(7)Å
c	14.8763(8)Å
β	91.382(3)°
Volume	2703.9(2)Å^3
Z	4
Calculated density	1.884Mg/m^3
F(000)	1504
Linear absorption coefficient μ	4.643mm^{-1}
Absorption correction	semi-empirical from equivalents
Max. and min. transmission	0.746 and 0.392
Unit cell determination	2.53° < Θ < 27.89°
	9978 reflections used at 100K
Data collection	
Temperature	100K
Diffractometer	Bruker APEX-II CCD
Radiation source	Incoatec microfocus sealed tube
Radiation and wavelength	MoK_{α}, 0.71073Å

Monochromator	multilayer monochromator
Scan type	ϕ and ω scans
Θ range for data collection	2.11 to 30.00°
Reflections collected / unique	65905 / 7832
Significant unique reflections	5681 with $I > 2\sigma(I)$
R(int), R(sigma)	0.1122, 0.0984
Completeness to Θ = 30.0°	99.3%
Refinement	
Refinement method	Full-matrix least-squares on F^2
Data / parameters / restraints	7832 / 384 / 0
Goodness-of-fit on F^2	1.037
Final R indices [$I > 2\sigma(I)$]	R1 = 0.0435, wR2 = 0.0898
R indices (all data)	R1 = 0.0731, wR2 = 0.0967
Extinction expression	none
Weighting scheme	$w = 1/[\sigma^2(F_o^2)+(aP)^2+bP]$ where $P = (F_o^2+2F_c^2)/3$
Weighting scheme parameters a, b	0.0248, 4.2449
Largest Δ/σ in last cycle	0.002
Largest difference peak and hole	1.842 and -2.243e/Å^3
Structure Solution Program	SHELXS-97[8]
Structure Refinement Program	SHELXL-2014/6[9]
CCDC number	2468749

Table S9. Selected bond lengths [Å] and angles [°] for **4b**

Re1-O1	1.680(3)
Re1-O2	1.959(3)
Re1-O4	1.963(3)
Re1-N12	2.113(4)
Re1-N32	2.080(4)
Re1-N1	2.144(4)
N11-N12	1.372(5)
O2-C21	1.355(5)
N31-N32	1.371(5)
O4-C41	1.362(6)

O1-Re1-O2	164.22(13)
N12-Re1-N32	169.35(14)
O4-Re1-N1	169.90(14)
C13-N12-N11	104.9(4)
C13-N12-Re1	127.4(3)
N11-N12-Re1	127.6(3)
C21-O2-Re1	129.5(3)
C33-N32-N31	105.7(4)
C33-N32-Re1	123.9(3)
N31-N32-Re1	129.7(3)
C41-O4-Re1	125.1(3)
C1-N1-Re1	164.2(4)
N1-C1-C2	177.3(5)

Crystal Structure Determination of 4c. All the measurements were performed using monochromatized Mo K_a radiation at 100K: $2(C_{24}H_{25}N_5O_5Re^+CF_3O_3S^-) \cdot C_2H_3N$, M_r 1638.57, monoclinic, space group P $2_1/n$, a = 13.0319(5)Å, b = 14.3383(5)Å, c = 15.6192(6)Å, b = 91.6045(18)°, V = 2917.39(19)Å^3, Z = 2, d_{calc} = 1.865g cm^{-3}, m = 4.315mm^{-1}. A total of 105201 reflections were collected (Q_{max} = 30.0°), from which 8478 were unique (R_{int} = 0.1176), with 6864 having I > 2s(I). The structure was solved by direct methods (SHELXS-97)[8] and refined by full-matrix least-squares techniques against F^2 (SHELXL-2014/6)[9]. The non-hydrogen atoms were refined with anisotropic displacement parameters without any constraints. The H atoms of the aromatic rings were put at the external bisectors of the X–C–C angles at C–H distances of 0.95Å and common isotropic displacement parameters were refined for the H atoms of the same ring. The H atoms of the methyl groups were refined with common isotropic displacement parameters for the H atoms of the same group and idealized geometries with tetrahedral angles, enabling rotations around the X–C bonds, and C–H distances of 0.98Å. For 431 parameters final *R* indices of R1 = 0.0389 and wR^2 = 0.0845 (GOF = 1.062) were obtained. The largest peak in a difference Fourier map was 1.739eÅ^{-3}.

Table S10. Crystal data and structure refinement for **4c**.

Crystal data		
Identification code		JSBM61
Empirical formula		$2(C_{24}H_{25}N_5O_5Re^+CF_3O_3S^-) \cdot C_2H_3N$
Formula weight		1638.57
Crystal description		block, brown
Crystal size		0.18 x 0.14 x 0.11mm
Crystal system, space group		monoclinic, P $2_1/n$
Unit cell dimensions:	a	13.0319(5)Å
	b	14.3383(5)Å
	c	15.6192(6)Å
	β	91.6045(18)°
Volume		2917.39(19)Å^3
Z		2
Calculated density		1.865Mg/m^3
F(000)		1612
Linear absorption coefficient μ		4.315mm^{-1}
Absorption correction		semi-empirical from equivalents
Max. and min. transmission		0.746 and 0.400
Unit cell determination		2.84° < Θ < 29.92°
		9848 reflections used at 100K
Data collection		
Temperature		100K
Diffractometer		Bruker APEX-II CCD
Radiation source		Incoatec microfocus sealed tube
Radiation and wavelength		MoK_α, 0.71073Å

Monochromator	multilayer monochromator
Scan type	ϕ and ω scans
Θ range for data collection	2.06 to 30.00°
Reflections collected / unique	105201 / 8478
Significant unique reflections	6864 with I > 2σ(I)
R(int), R(sigma)	0.1176, 0.0631
Completeness to Θ = 30.0°	99.7%
Refinement	
Refinement method	Full-matrix least-squares on F^2
Data / parameters / restraints	8478 / 431 / 0
Goodness-of-fit on F^2	1.062
Final R indices [I > 2σ(I)]	R1 = 0.0389, wR2 = 0.0799
R indices (all data)	R1 = 0.0515, wR2 = 0.0845
Extinction expression	none
Weighting scheme	$w = 1/[\sigma^2(F_o^2)+(aP)^2+bP]$ where $P = (F_o^2+2F_c^2)/3$
Weighting scheme parameters a, b	0.0388, 0.0000
Largest Δ/σ in last cycle	0.003
Largest difference peak and hole	1.739 and -2.030e/Å^3
Structure Solution Program	SHELXS-97[8]
Structure Refinement Program	SHELXL-2014/6[9]
CCDC number	2468750

Table S11. Selected bond lengths [Å] and angles [˚] for **4c**

Re1-O1	1.695(2)
Re1-O2	1.950(2)
Re1-O4	1.948(2)
Re1-N12	2.076(3)
Re1-N32	2.130(3)
Re1-N1	2.161(3)
N11-N12	1.375(4)
O2-C21	1.360(4)
C24-O27	1.376(4)
O27-C27	1.416(5)
N31-N32	1.368(4)
O4-C41	1.360(4)
C44-O47	1.375(4)
O47-C47	1.428(4)

O1-Re1-O4	163.26(10)
N12-Re1-N32	167.75(11)
O2-Re1-N1	170.35(10)
C13-N12-N11	105.5(3)
C13-N12-Re1	125.4(2)
N11-N12-Re1	128.9(2)
C21-O2-Re1	126.6(2)
O27-C24-C23	115.5(3)
O27-C24-C25	124.2(3)
C24-O27-C27	117.5(3)
C33-N32-N31	106.3(3)
C33-N32-Re1	126.7(2)
N31-N32-Re1	127.0(2)
C41-O4-Re1	130.3(2)
O47-C44-C43	125.3(3)
O47-C44-C45	114.5(3)

C44-O47-C47	116.8(3)
C1-N1-Re1	165.9(3)
N1-C1-C2	177.2(4)

Solid state structure of [$ReOCl_2$(**L2e**)(py)] (**5e**).

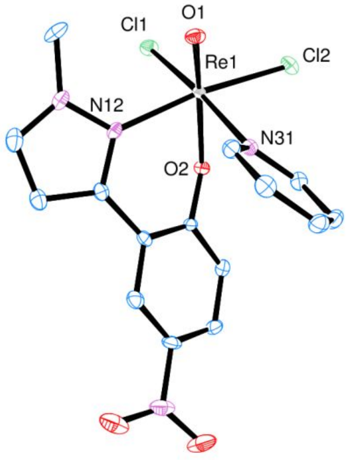

Figure S25. Molecular views (50% level) of complex **5e** (H atoms omitted for clarity).

Crystal Structure Determination of 5e. All the measurements were performed using monochromatized Mo K_{α} radiation at 100K: $C_{15}H_{13}Cl_2N_4O_4Re$, M_r 570.39, monoclinic, space group P 2_1/c, a = 8.8110(5)Å, b = 17.7239(11)Å, c = 11.8115(7)Å, β = 107.593(2)°, V = 1758.27(18)$Å^3$, Z = 4, d_{calc} = 2.155g cm^{-3}, μ = 7.245mm^{-1}. A total of 38885 reflections were collected (Θ_{max} = 30.0°), from which 5135 were unique (R_{int} = 0.0562), with 4532 having I > 2σ(I). The structure was solved by direct methods (SHELXS-97)[8] and refined by full-matrix least-squares techniques against F^2 (SHELXL-2014/6)[9]. The non-hydrogen atoms were refined with anisotropic displacement parameters without any constraints. The H atoms of the aromatic rings were put at the external bisectors of the C–C–X angles at C–H distances of 0.95Å and common isotropic displacement parameters were refined for the H atoms of the same ring. The H atoms of the methyl group were refined with a common isotropic displacement parameter and idealized geometry with tetrahedral angles, enabling rotation around the C–N bond, and C–H distances of 0.98Å. The largest peaks in a final difference Fourier map (1.73 - 2.20e$Å^{-3}$) were in the vicinity (0.80 - 1.38Å) of the Re atom. For 240 parameters final *R* indices of R1 = 0.0309 and wR^2 = 0.0785 (GOF = 1.091) were obtained.

Table S12. Crystal data and structure refinement for **5e**.

Crystal data	
Identification code	JSJ6
Empirical formula	$C_{15}H_{13}Cl_2N_4O_4Re$
Formula weight	570.39
Crystal description	block, green
Crystal size	0.22 x 0.15 x 0.11mm
Crystal system, space group	monoclinic, P 2_1/c
Unit cell dimensions: a	8.8110(5)Å
b	17.7239(11)Å
c	11.8115(7)Å
β	107.593(2)°
Volume	1758.27(18)$Å^3$
Z	4
Calculated density	2.155Mg/m^3
F(000)	1088
Linear absorption coefficient μ	7.245mm^{-1}
Absorption correction	semi-empirical from equivalents
Max. and min. transmission	0.746 and 0.393
Unit cell determination	2.68° < Θ < 30.16°
	9358 reflections used at 100K
Data collection	
Temperature	100K
Diffractometer	Bruker APEX-II CCD
Radiation source	Incoatec microfocus sealed tube
Radiation and wavelength	MoK_{α}, 0.71073Å
Monochromator	multilayer monochromator
Scan type	ϕ and ω scans
Θ range for data collection	2.14 to 30.00°
Reflections collected / unique	38885 / 5135
Significant unique reflections	4532 with I > 2σ(I)
R(int), R(sigma)	0.0562, 0.0530
Completeness to Θ = 30.0°	100.0%
Refinement	
Refinement method	Full-matrix least-squares on F^2
Data / parameters / restraints	5135 / 240 / 0
Goodness-of-fit on F^2	1.091
Final R indices [I > 2σ(I)]	R1 = 0.0309, wR2 = 0.0777
R indices (all data)	R1 = 0.0338, wR2 = 0.0785
Extinction expression	none
Weighting scheme	$w = 1/[\sigma^2(F_o^2)+(aP)^2+bP]$ where $P = (F_o^2+2F_c^2)/3$
Weighting scheme parameters a, b	0.0345, 0.0000
Largest Δ/σ in last cycle	0.001
Largest difference peak and hole	2.198 and -1.637e/$Å^3$
Structure Solution Program	SHELXS-97[8]
Structure Refinement Program	SHELXL-2014/6[9]
CCDC number	2468752

Table S13. Selected bond lengths [Å] and angles [°] for **5e**

Re1-O1	1.682(2)
Re1-O2	1.9695(19)
Re1-N12	2.124(3)
Re1-N31	2.165(3)
Re1-Cl1	2.3420(8)
Re1-Cl2	2.3615(8)
O2-C21	1.346(4)

O1-Re1-O2	164.29(10)
N31-Re1-Cl1	172.72(6)
N12-Re1-Cl2	169.75(7)
N11-N12-C13	105.7(3)
N11-N12-Re1	127.30(19)
C13-N12-Re1	126.8(2)
C21-O2-Re1	131.33(18)
C32-N31-C36	118.5(3)
C32-N31-Re1	121.7(2)
C36-N31-Re1	119.7(2)

Crystal Structure Determination of 6d'. All the measurements were performed using monochromatized Mo K_a radiation at 100K: $C_{21}H_{19}Br_2N_4O_4Re$, M_r 737.42, orthorhombic, space group P n a 2_1, a = 14.5353(7)Å, b = 18.2228(9)Å, c = 8.2343(4)Å, V = 2181.05(18)Å^3, Z = 4, d_{calc} = 2.246g cm^{-3}, m = 9.273mm^{-1}. A total of 86282 reflections were collected (Q_{max} = 30.0°), from which 6370 were unique (R_{int} = 0.0833), with 6048 having I > 2s(I). The structure was solved by direct methods (SHELXS-97)[8] and refined by full-matrix least-squares techniques against F^2 (SHELXL-2014/6)[9]. The non-hydrogen atoms were refined with anisotropic displacement parameters without any constraints. The absolute configuration was established by anomalous dispersion effects in the diffraction measurements on the crystal. Since racemic twinning was detected a twin matrix (-1 0 0 / 0 -1 0 / 0 0 -1) was applied and a scale factor was refined [0.109(8)] between the two unequal components. The H atoms of the ring atoms were put at the external bisectors of the C–C–X angles at C–H distances of 0.95Å and common isotropic displacement parameters were refined for the H atoms of the same ring. The H atoms of the methyl groups were refined with common isotropic displacement parameters for the H atoms of the same group and idealized geometries with tetrahedral angles, enabling rotations around the C–X bonds, and C–H distances of 0.98Å. For 300 parameters final *R* indices of R1 = 0.0239 and wR^2 = 0.0484 (GOF = 1.042) were obtained. The largest peak in a difference Fourier map was 1.016eÅ^{-3}.

Table S14. Crystal data and structure refinement for **6d'**.

Crystal data	
Identification code	JSJ16
Empirical formula	$C_{21}H_{19}Br_2N_4O_4Re$
Formula weight	737.42
Crystal description	block, blue
Crystal size	0.13 x 0.08 x 0.06mm
Crystal system, space group	orthorhombic, P n a 2_1
Unit cell dimensions: a	14.5353(7)Å
b	18.2228(9)Å
c	8.2343(4)Å
Volume	2181.05(18)Å^3
Z	4
Calculated density	2.246Mg/m^3
F(000)	1400
Linear absorption coefficient μ	9.273mm^{-1}
Absorption correction	semi-empirical from equivalents
Max. and min. transmission	0.747 and 0.428
Unit cell determination	2.64° < Θ < 30.09°
	9540 reflections used at 100K
Data collection	

Temperature	100K
Diffractometer	Bruker APEX-II CCD
Radiation source	Incoatec microfocus sealed tube
Radiation and wavelength	MoK_{α}, 0.71073Å
Monochromator	multilayer monochromator
Scan type	ϕ and ω scans
Θ range for data collection	2.64 to 30.00°
Reflections collected / unique	86282 / 6370
Significant unique reflections	6048 with $I > 2\sigma(I)$
R(int), R(sigma)	0.0833, 0.0380
Completeness to Θ = 30.0°	99.9%
Refinement	
Refinement method	Full-matrix least-squares on F^2
Data / parameters / restraints	6370 / 300 / 1
Goodness-of-fit on F^2	1.042
Final R indices [$I > 2\sigma(I)$]	R1 = 0.0239, wR2 = 0.0477
R indices (all data)	R1 = 0.0267, wR2 = 0.0484
Absolute structure parameter	0.109(8)
Extinction expression	none
Weighting scheme	$w = 1/[\sigma^2(F_o^2)+(aP)^2+bP]$ where $P = (F_o^2+2F_c^2)/3$
Weighting scheme parameters a, b	0.0159, 1.8192
Largest Δ/σ in last cycle	0.002
Largest difference peak and hole	1.016 and -0.853e/Å^3
Structure Solution Program	SHELXS-97[8]
Structure Refinement Program	SHELXL-2014/6[9]
CCDC number	2468751

Table S15. Selected bond lengths [Å] and angles [˚] for **6d’**

Re1-O1	1.695(3)
Re1-O10	1.954(3)
Re1-O2	2.026(3)
Re1-O4	1.988(3)
Re1-N12	2.124(4)
Re1-N32	2.127(4)
O10-C10	1.421(6)
O2-C21	1.345(6)
O4-C41	1.341(5)

O1-Re1-O2	164.54(15)
O4-Re1-N12	166.37(15)
O10-Re1-N32	161.41(14)
O2-Re1-O4	88.08(13)
N12-Re1-N32	98.38(15)
C10-O10-Re1	126.0(3)
N11-N12-C13	106.0(4)
N11-N12-Re1	126.2(3)
C13-N12-Re1	126.8(3)
C21-O2-Re1	124.2(3)
N31-N32-C33	106.1(4)
N31-N32-Re1	128.1(3)
C33-N32-Re1	124.0(3)
C41-O4-Re1	127.1(3)

References

[1] Traar, P.; Schachner, J. A.; Steiner, L.; Sachse, A.; Volpe, M.; Mösch-Zanetti, N. C. Oxorhenium(V) Complexes with Pyrazole Based Aryloxide Ligands and Application in Olefin Epoxidation. *Inorg. Chem.* **2011**, *50*, 1983–1990.

[2] Zwettler, N.; Schachner, J. A.; Belaj, F.; Mösch-Zanetti, N. C. Oxorhenium(V) Complexes with Phenolate–Pyrazole Ligands for Olefin Epoxidation Using Hydrogen Peroxide. *Inorg. Chem.* **2014**, *53*, 12832–12840.

[3] Pleier, A.-K.; Glas, H.; Grosche, M.; Sirsch, P.; Thiel, W. R. Microwave Assisted Synthesis of 1-Aryl-3-dimethylaminoprop-2-enones: A Simple and Rapid Access to 3(5)-Arylpyrazoles. *Synthesis* **2001**, 55–62.

[4] a) Nawrot-Modranka, J.; Kostka, K. Studies of chromone derivatives. Part XV. Reactions of chromone and its derivatives with methylhydrazine. *Pol. J. Chem.* **1988**, *62*, 417; b) Catalan, J.; Fabero, F.; Claramunt, R. M.; Maria, M. D. S.; Focesfoces, M. D. C.; Cano, F. H.; Martinezripoll, M.; Elguero, J.; Sastre, R. New Ultraviolet Stabilizers: - 3-(2'-Hydroxyphenyl)pyrazole and 5-(2'-Hydroxyphenyl)pyrazole. *J. Am. Chem. Soc.* **1992**, *114*, 5039–5048;

[5] Akram, M. O.; Bera, S.; Patil, N. T. A facile strategy for accessing 3-alkynylchromones through gold-catalyzed alkynylation/cyclization of o-hydroxyarylenaminones. *Chem. Commun.* **2016**, *52*, 12306–12309.

[6] Balbi, A.; Anzaldi, M.; Macciò, C.; Aiello, C.; Mazzei, M.; Gangemi, R.; Castagnola, P.; Miele, M.; Rosano, C.; Viale, M. Synthesis and biological evaluation of novel pyrazole derivatives with anticancer activity. *Eur. J. Med. Chem.* **2011**, *46*, 5293–5309.

[7] Praveen Kumar, V.; Gajendra Reddy, R.; Vo, D. D.; Chakravarty, S.; Chandrasekhar, S.; Grée, R. Synthesis and neurite growth evaluation of new analogues of honokiol, a neolignan with potent neurotrophic activity. *Bioorg. Med. Chem. Lett.* **2012**, *22*, 1439–1444.

[8] Sheldrick, G. M. A short history of SHELX. *Acta Crystallogr., Sect. A: Found.* **2008**, 112–122.

[9] Sheldrick, G. M. Crystal structure refinement with SHELXL. *Acta Crystallogr., Sect. C: Cryst. Struct. Chem.* **2015**, 3–8.
